# Environmental influences on the distribution and ecology of the fluke intermediate host *Galba truncatula*: a systematic review

**DOI:** 10.1017/S0031182024000957

**Published:** 2024-09

**Authors:** Christopher David Smith, Eric René Morgan, Rhys Aled Jones

**Affiliations:** 1 Department of Life Sciences, Aberystwyth University, Penglais, Aberystwyth, UK; 2 School of Biological Sciences, Queen's University Belfast, Belfast, UK.

**Keywords:** *Calicophoron daubneyi*, climate change, ecology, environment, *Fasciola hepatica*, *Galba truncatula*, habitat

## Abstract

*Galba truncatula* is one of the most distributed intermediate hosts of *Fasciola hepatica* across Europe, North Africa and South America. Therefore, understanding the environmental preferences of this species is vital for developing control strategies for fascioliasis and other trematodes such as *Calicophoron daubneyi*. This systematic literature review evaluates the current understanding of the snail's environmental preferences to identify factors which might aid control and areas where further research is needed. Searches were conducted using Google Scholar and PubMed and included papers published up to August 2023. After filtration, 198 papers with data from 64 countries were evaluated, and data regarding habitat type and habitat pH were noted, along with any other information pertaining to the snail's environmental preferences. The results show that *G. truncatula* can survive in a diverse range of climates and habitats, generally favours shallow slow-moving water or moist bare mud surfaces, temperatures between 10 and 25°C and was found in habitats with a water pH ranging from 5.0 to 9.4. However, there is limited understanding of the impact of several factors, such as the true optimum pH and temperature preferences within the respective tolerance limits or the reason for the snail's apparent aversion to peatland. Further research is needed to clarify the impact of biotic and abiotic factors on the snail to create robust risk assessments of fluke infection and assess opportunities for environmental control strategies, and for predicting how the snail and fluke transmission may be impacted by climate change.

## Graphical abstract

**Figure:**
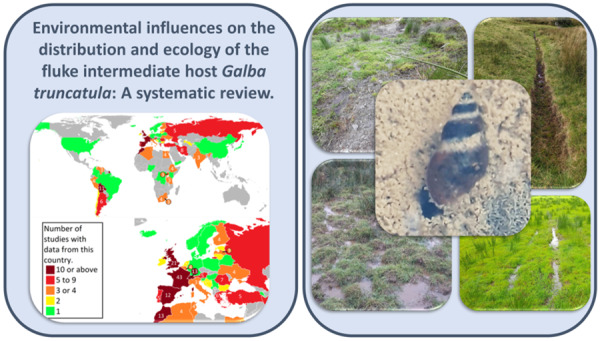

## Introduction

*Galba truncatula* is an amphibious lymnaeid snail which is best known for being the intermediate host of several trematode parasites (Bargues *et al*., 2003). The species was first described as *Buccinum truncatulum* (Müller, 1774), though through the 1900s it was commonly referred to as *Limnaea truncatula*, *Lymnaea truncatula* or *G. truncatula* due to unclear physiology-based taxonomy, with the latter eventually becoming preferred.

The most notable parasite carried by *G. truncatula* is the liver fluke *Fasciola hepatica*, which can infect livestock, wildlife and humans as the definitive hosts and has been estimated to cause €2.5 billion in damage to the global agricultural industry per annum (European Commission, 2012) and £111 million in the UK alone (Charlier *et al*., 2020). Additionally, between 2 and 17 million people are thought to be infected with *F. hepatica* (Hopkins, 1992; WHO, 2020), with infection rates in hyperendemic regions possibly reaching as high as 53–100% in the Aymara people of Bolivia (Hillyer *et al*., 1992). *Galba truncatula* is also the intermediate host of the rumen fluke *Calicophoron daubneyi*, a parasite traditionally associated with tropical regions which has increased in prevalence in Europe over the last few decades (Mage *et al*., 2002; Huson *et al*., 2017; Jones *et al*., 2017*a*).

The primary control method applied in livestock for both *F. hepatica* and *C. daubneyi* is anthelmintic treatment, and, as such, the growing incidence of anthelmintic resistance is a significant threat to the successful control of these parasites. Resistance towards triclabendazole, the primary drug used to treat *F. hepatica* (and the only 1 active against the most damaging immature stages), has occurred in at least 11 countries since it was first reported in the 1990s (Kelley *et al*., 2016; CDC, 2020), with reduced efficacy being reported at most surveyed sheep farms in 1 UK-based study (Kamaludeen *et al*., 2019). Resistance to other drugs used to treat adult *F. hepatica* infections (including closantel, albendazole, clorsulon and nitroxinil) has also been reported on farms (Fairweather and Boray, 1999; Martínez-Valladares *et al*., 2014; Novobilský and Höglund, 2015; Kelley *et al*., 2016).

Anthelmintic resistance is yet to be detected in *C. daubneyi*. However, currently only a single drug, oxyclozanide, is widely available to treat *C. daubneyi* (Havrdová *et al*., 2023), so future resistance development could have a catastrophic impact on *C. daubneyi* control. Anthelmintic resistance is partially driven by inappropriate use of anthelmintics, with more targeted treatments recommended but difficult to apply in practice given limited uptake and performance of available diagnostic tests (Charlier *et al*., 2008; Alvarez Rojas *et al*., 2014). The mechanisms of drug resistance are not fully understood which further hampers fluke control.

Given the current issues with diagnosis and treatment, and the growing threat of anthelmintic resistance, there is increasing interest in control methods that focus on non-chemical preventative strategies. By reducing levels of infection in this way, the need for anthelmintic drugs is also reduced, which could slow down the spread of resistance. Preventative strategies may also be beneficial to farmers in less economically developed countries, where the price of anthelmintic drugs is a relatively greater burden. Understanding the ecology of the intermediate snail hosts is key to developing effective preventative strategies. For example, by furthering understanding of which environmental factors favour snail populations, and how they respond to various changes in their habitat, efficient identification of snail habitats and improved strategies for preventing livestock infection through evasive grazing can be developed.

More resilient preventative strategies are especially needed given the potential impact of climate change. Rising temperatures and changes to seasonal weather patterns could have a positive or negative impact on snail populations depending on the optimal conditions for each snail species. This, in turn, will impact the ability of the parasite to propagate given the availability of intermediate host species, and thus alter infection levels and disease risk to livestock and people. Predictions by Fox *et al*. (2011) suggest that the risk of fascioliasis is likely to greatly increase in the future in the UK, but with some areas of reduced or fluctuating infection rates based on erratic weather and local conditions. As seasonal weather patterns change and become less predictable, understanding which conditions are favourable for the main intermediate host species will be vital to understanding how the changing climate may affect the transmission of trematode infections.

*Galba truncatula* is the main intermediate host of *F. hepatica* in Europe and is also a prominent species in many other parts of the world including North Africa and South America (Mas-Coma *et al*., 2001; Caron *et al*., 2014, 2017). As such, there is a substantial amount of research analysing various aspects of the snail's ecology. These studies focus on a wide range of topics, examining how various aspects of the snails' environment impact their abundance, biology and behaviour. However, while these pieces of research are important individually, they are mostly focused on a specific region or country. Collating ecological information regarding *G. truncatula* snails across a wide range of regions and climates can provide valuable information, allowing variations in snail preferences and ecology to be assessed and the impact of future changes predicted beyond those historically experienced within a given region. To that end, the aim of this study was to systematically collate and assess the current state of knowledge of the environmental preferences of *G. truncatula* as described within the scientific literature and evaluate its certainty. This includes evaluating findings from older studies and comparing them with more modern data to evaluate discrepancies and how our understanding of the snail has changed. More specifically, to gain insight into:

1. The preferred habitats of *G. truncatula* and how they are defined.
2. The impact of large-scale climatic factors and local ecological factors on *G. truncatula* populations.
3. Environmental factors and human activities that impact *G. truncatula* ecology.
4. How adaptable *G. truncatula* is to changes to their environment over the short and long term, and finally
5. How *G. truncatula* ecology impacts the transmission and spread of livestock parasites.

## Materials and methods

### Protocol and registration

The systematic review was conducted using Preferred Reporting Items for Systematic Reviews and Meta Analyses (PRISMA) guidelines (Page *et al*., 2021), which was confirmed using a checklist (Supplementary Table S1). The protocol has not been published separately and the review was not registered prior to publication.

### Search strategy

The articles for this review were primarily found using 2 searches on Web of Science using the terms ‘*Galba truncatula* habitat’ and ‘*Lymnaea truncatula* habitat’. In addition to these 2 primary searches, a number of supplementary searches were conducted. The primary search terms were searched on Google Scholar, and then ‘*Galba truncatula*’ and ‘*Lymnaea truncatula*’ were searched on both Google Scholar and Web of Science followed by the following list of terms:

Ecology, pH, Acidity, Salinity, Altitude, Animal Transport, Temperature, Germany, Finland, Montenegro, India, Romania, Bulgaria, Greece, Armenia, Azerbaijan, Serbia, Lithuania, Latvia, Estonia, Israel, Belarus, Georgia, Libya and Croatia.

Countries were used as a search term if no studies were found in prior searches that physically found populations of *G. truncatula* in that country, but populations had been identified in neighbouring countries. In some cases, *G. truncatula* data had been used in other studies (such as in literature reviews), but no populations had been physically identified by those papers. Not all countries which met the criteria above were used as search terms due to time constraints.

In addition to these searches, specific studies were identified based on citations in other papers. All results from the Web of Science searches were considered for inclusion, along with the first page of results from those on Google Scholar (with results sorted by relevance).

For the 2 primary searches, any papers which could not be accessed were requested using the document request system at Aberystwyth University. This was not done for inaccessible papers during the supplementary searches. All papers considered were published between 1st of January 1900 and 31st of August 2023. Papers published after the 31st of August 2023 could be cited in the discussion section if they contained relevant information, but they were not included in any of the summary statistics. In the 2 primary searches, all accessible papers were read in full to determine if they met the eligibility criteria below and were translated into English using Google Translate if needed. For the supplementary searches, the titles and abstracts were scanned to determine inclusion and only English language papers were considered. The 1 exception to this was Rondelaud (1983) because it was an important paper concerning snail movement. The searches were conducted between March and August 2023.

The original authors were not contacted for additional information and no attempt was made to access unpublished data.

### Eligibility criteria

To be included in this review, studies had to contain information related to *G. truncatula* ecology as defined by the inclusion criteria (see Table 1) for at least part of the study. Studies which also focused on other species were included, but they had to meet the inclusion criteria and focus on *G. truncatula* as well. One researcher (Christopher David Smith) conducted the searches and screened the studies for eligibility.

**Table 1.** List of inclusion and exclusion criteria for studies in this systematic literature review, along with a list of study types which were excluded

Inclusion criteria	Exclusion criteria
Field or laboratory studies which focused on the impact of environmental conditions on the physiology, abundance or presence of the snail. Ecological factors could include temperature, precipitation, altitude, physio-chemical properties of the snail habitat (e.g. pH, salinity, soil type), competition with other species and the unintentional consequences of human activity (e.g. pollution).	Studies which solely focused on species other than *G. truncatula* and included no information on the ecology or environmental preferences of *G. truncatula* (as defined by the inclusion criteria). This includes studies solely focused on the parasites of *G. truncatula*, the definitive hosts of these parasites, as well as the predators and competitors of *G. truncatula*.
Studies which focused on identifying the presence or distribution of *G. truncatula* across a geographic area or areas, either through surveys which identified *G. truncatula* morphologically or molecularly, or *via* literature reviews. This includes surveys which focused on identifying mollusc species in general which identified the presence of *G. truncatula*. Also included were genetic studies which identified cases in which *G. truncatula* had been misidentified as another species, or vice versa, as these studies provide evidence of the geographic distribution of *G. truncatula*.	Laboratory experiments which use *G. truncatula* to study the parasites of the snail but include no information about the environmental preferences or ecology of the snail as defined by the inclusion criteria. This includes studies which studied the impact of environmental conditions such as temperature on the ability of the parasites to infect *G. truncatula* in laboratory conditions unless the impact of these conditions on the snail was also studied.
Field and laboratory studies and literature reviews which included information on the mobility of *G. truncatula* within or between habitats, including the influence of other animals and human activity.	Studies which mention information about the environmental preferences and ecology of *G. truncatula* in the introduction, discussion or conclusions drawn from other studies.
Field and/or laboratory studies which examined how aspects of the ecology of *G. truncatula* may influence its population structure.	Studies which focused on historical or fossil populations of *G. truncatula*.
Field and/or laboratory studies which focused on the differences in infection rates and responses to infection between different populations of *G. truncatula*.	Genetic studies focused on the taxonomy of *G. truncatula* and other molluscs.
Studies which met the above criteria but also contained information on other species.	Studies focused on measuring the impact of molluscicides or testing potential new molluscicides

Please note that if a study met the inclusion criteria for at least part of the paper, it was included.

### Risk of bias

After screening, risk of bias was assessed using the MMAT (Mixed Methods Appraisal Tool) (Nha Hong *et al*., 2018). This tool is designed for simultaneously appraising studies using a range of methodologies, including qualitative, quantitative and mixed-methods studies. One researcher (Christopher David Smith) performed the risk of bias appraisal. This assessment recommends at least 2 appraisers, but 1 was used to ensure a standard approach given the large number of different study types in this review.

### Data extraction

Information regarding the date of publication and the country or countries of origin of the *G. truncatula* data used in the study was then extracted from the papers. The country of origin of the data was used rather than the country the study took place because some studies used data from *G. truncatula* populations located elsewhere. As such, this was a better representation of the species' maximum potential geographic range.

The papers were then sorted based on the type of study (see Table 2). Next, the papers were screened for any descriptions of *G. truncatula* habitats. This was done in 2 phases: First was the ‘Per Study Count’ which counted whether a type of habitat was mentioned by the study. Second was the ‘Individual Habitat Count’ which counted the total number of each type of habitat that *G. truncatula* was found in across all the studies. This was done to compare whether the habitats investigated by the largest number of studies are the most common habitats of the snail. The comparison between the counts would only give a rough estimate because not all studies which described the habitats where the snails were found gave the precise numbers of each habitat. The categories for each type of habitat were largely drawn from the studies themselves. This was done for 2 reasons. Firstly, to demonstrate the fullest possible range of habitats that *G. truncatula* inhabits, including those which may be rare. Secondly, to investigate the way studies categorize the different types of habitats and identify potential issues such as a lack of clarity in habitat definition.

**Table 2.** Types of study and definitions for each study type

Type of study	Definition
Lab-based study	Almost all the research was conducted in a laboratory. There may be a brief section in which they discuss the collection of snails from a natural habitat, but the rest of the research is focused on procedures within the laboratory.
Field and lab study	Both field observations or procedures and those conducted in the laboratory made up a large part of the overall methodology.
Field experiment	These studies are conducted in the field, but rather than simply collecting data on already existing habitats they focus on constructed situations to test the impact of certain factors. Alternatively, they may focus on testing whether an approach for detecting the presence of *G. truncatula* can be successfully applied in the field.
Small-scale survey	These studies focused on recording the presence and/or abundance of the snail. Often these studies also record 1 or more factors linked to the snail's abundance or presence to see if these have a significant impact. The difference between small and large surveys was defined as whether 100 or more sites were investigated over the course of the study.
Large-scale survey	A survey in which 100 or more sites were investigated (see above for definition of survey).
Literature review	A paper which the researchers did not conduct any direct observations or experiments on *G. truncatula*, instead collecting and analysing data from previous studies.

It should be noted that there were a few instances in which studies presented the numbers of each habitat type but were still excluded from the individual habitat count. The most notable was (Vignoles *et al*., 2022) which investigated the decline in the snail abundance using the same habitats investigated in 2 prior studies. As such, only the 2 prior studies were included in the counts. After both counts were completed, the habitat types were ranked separately for each count, and the rankings compared. Any habitat types which were counted the same number of times in either count were given the same rank.

**Figure 1.**
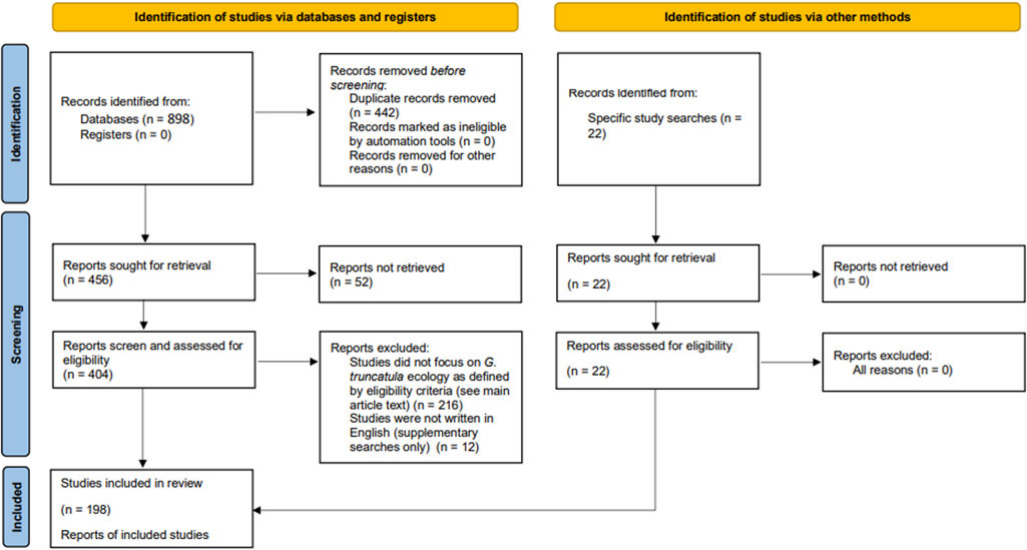
PRISMA flow chart showing the study selection process for this literature review.

**Figure 2.**
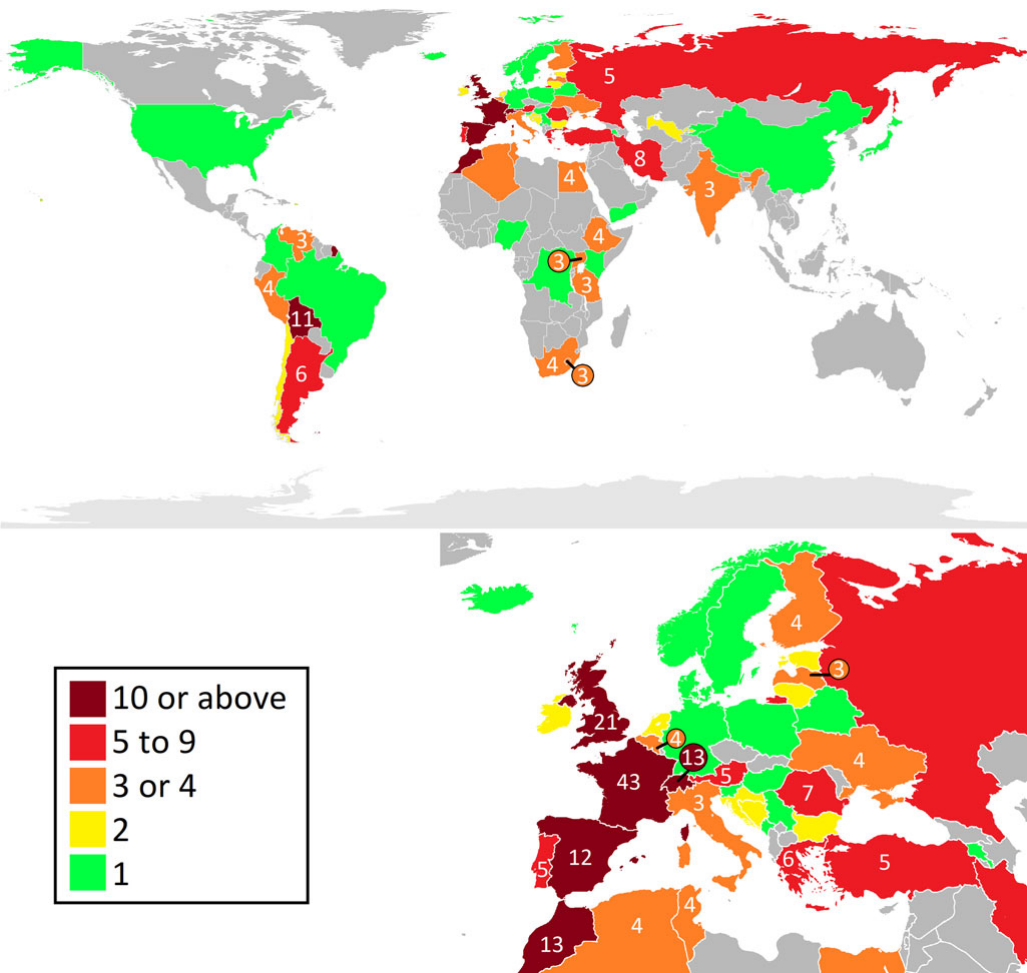
Map showing the number of studies used *G. truncatula* data from each country. Countries were broken into categories based on the number of studies, shown by the colour scale, while the number labels show the exact number of studies for each country. Based on public domain maps from Wikipedia.

The studies were also examined for information regarding the pH of water and soil in the snail's habitats. If the mean, maximum and minimum pH were stated, they were recorded. The studies were further categorized based on whether *G. truncatula* presence or abundance was correlated with pH, and were assigned either ‘Positive’, ‘Negative’ or ‘No Preference or Neutral’. Any data missing from these summary statistics were assumed to not be recorded by the study in question. Figures 1 and 2 were created in Microsoft Word and Microsoft Paint, respectively, while all other were figures created from the extracted data that were made in R studio using R version 4.2.1 (R Core Team, 2022) and the ggplot2 package (v3.4.5, Wickham, 2023).

Finally, the papers were also examined, and their aims, methodology and findings related to *G. truncatula* ecology were recorded and compared qualitatively. A specific system for assessing certainty was not used, as these tend to focus on the impact of specific treatments. Instead, certainty criteria such as the consistency of terminology and level of detail were assessed qualitatively and are discussed in the Results and Discussion sections.

## Results

### Search results

The search terms gave a total of 898 results, of which 176 were used in this systematic literature review, along with an additional 22 studies which were found outside the search criteria as described above, resulting in 198 studies being used for the summary statistics. Figure 1 shows a flow chart of the study design. Not all these studies are directly cited in the text but are included in the reference list. The full list of the studies included, along with their summary statistics, can be found in Supplementary Table S4.

### Assessment of bias

In total, 156 of the 198 studies used were classed as ‘Qualitative Descriptive’, as they focused on assessing natural populations of *G. truncatula* rather than performing experiments to test specific hypotheses. The next most common study type was ‘Quantitative Non-Randomized’ (28), then ‘Quantitative Randomized Controlled’ (9) and ‘Mixed Model’ (5, all of which were Quantitative Non-Randomized/Descriptive). None of the studies were Qualitative. The risk of non-response bias was difficult to judge in most Quantitative Descriptive studies. None of the studies directly reported on these data, and it was difficult to assess whether this would apply to most ecological surveys, so it was usually marked as ‘Can't tell’ (121/161). Furthermore, 50 of the Quantitative Descriptive studies were marked as ‘Can't tell’ for statistical analysis appropriate to the research question. This was mostly due to several studies having extremely limited statistical analysis (for instance, if they simply reported the number of molluscs present at each site). The full details are shown in Supplementary Table S3.

### Country of origin of data

In total, *G. truncatula* data from 64 countries were used in the studies reviewed. Data from France appeared in the most studies at 43, while data from most countries appeared in only 1 paper each (see Fig. 2). Outside of Europe and North Africa, data from Bolivia, Iran and Argentina were present in the highest number of studies.

### Study and habitat type

**Figure 3.**
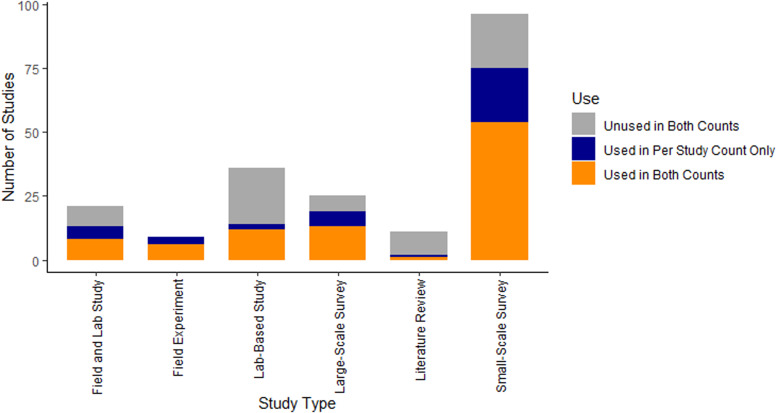
The number of each type of study included in the literature review. The sum of all 3 sections of each bar shows the total number of each type included in the study.

The most common type of study was ‘Small-Scale Surveys’ at 96, while the least common was ‘Literature Reviews’ at 11. Of the 198 papers, 131 were used in the Per Study Count and 94 were used in the Individual Habitat Count. Field Experiments had the highest proportion of studies included in both counts, while literature reviews had the lowest proportion (see Fig. 3). A total of 68 different habitat types were identified based on the information in the studies reviewed. However, 11 of these habitat types were only found in studies which did not detail how many of these habitats *G. truncatula* inhabited. As such, they were excluded from the Individual Habitat Count, and were not considered when the rankings were compared.

**Figure 4.**
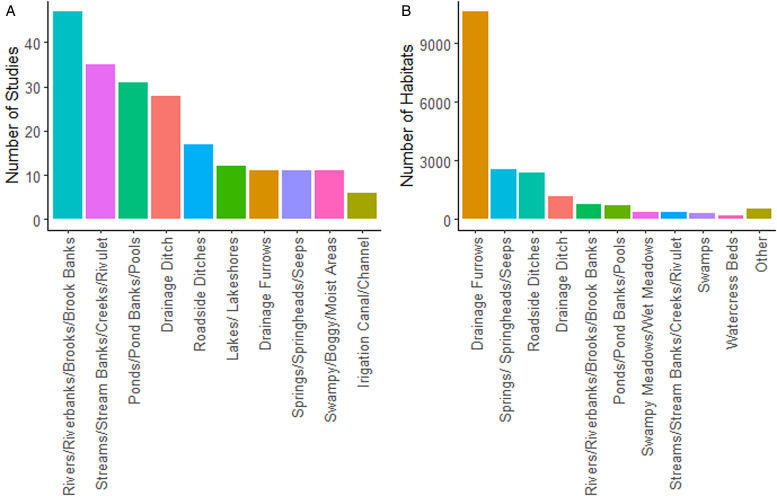
Bar charts showing the top 10 results of the Per Study (A) and Individual Habitat **(**B**)** counts. Figure (A) shows the number of studies in which *G. truncatula* was found in each habitat type. Figure (B) shows the number of each habitat type which were recorded across all the studies examined.

The habitat which was highest in the per study count was ‘Rivers/Riverbanks/Brooks/Brook Banks’, which was found to be a *G. truncatula* habitat in 47 studies (see Fig. 4A), while the majority of the habitat types (33) were only stated in 1 study. ‘Drainage Furrows’ had by far the largest number in the Individual Habitat Count at 10 626. The next most common habitats were ‘Springs/Springheads/Seeps’ at 2523 and ‘Roadside Ditches’ at 2366 (see Fig. 4B). Seven of the top 10 most common habitat types in the Per Study count were also in the top 10 of the Individual Habitat Count.

**Figure 5.**
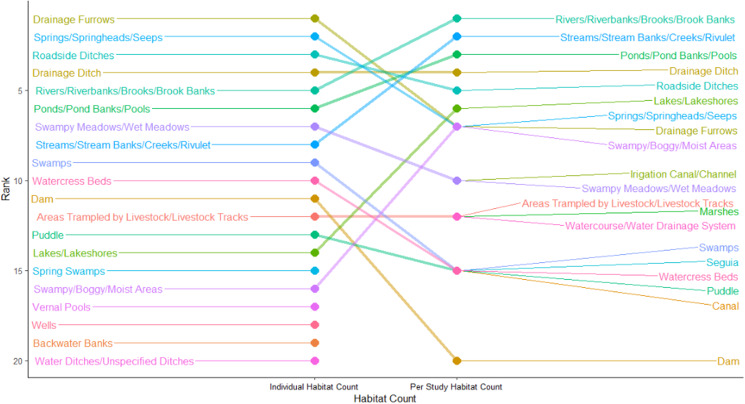
Slope graph showing a comparison of the top 20 ranked habitat types in the Per Study and Individual Habitat Counts.

When the ranking of the 2 counts is compared (see Fig. 5), it seems that the rank difference tends to be small in more common habitats, and then becomes more unpredictable. The rarest habitats also tend to be closely ranked, especially if they represent a single habitat which appeared in 1 study. However, rarer habitats also tend to be excluded from the individual habitat count, as it was more common that studies would not report how many of these habitats were sampled. Three habitat types, ‘Drainage Ditches’, ‘Areas Trampled by Livestock/Livestock Tracks’ and ‘Water Ditches/Unspecified Ditches’ were ranked equally in both counts while ‘Marshes’ had the largest difference (as this category was 23 ranks lower in the individual count).

### pH preferences

**Figure 6.**
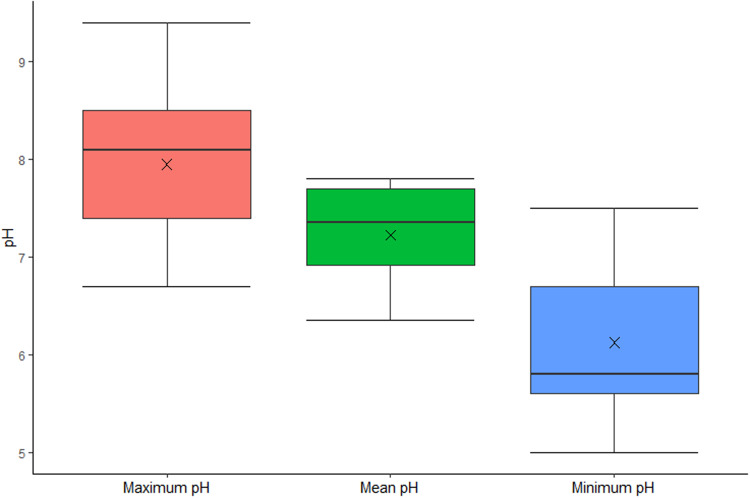
Box and Whisker plot showing the range of pH values found in the water of *G. truncatula* habitats.

In total, 33 studies provided some information regarding the pH of *G. truncatula* habitats (a full list is provided in Supplementary Table S5). Of these, 31 used the pH of the water, 1 used both soil and water (though they did not provide the minimum, maximum or mean) and 1 did not state which was used, and as such was excluded from the summary statistics. As only 1 study used soil pH, a comparison with the water pH results was not possible. The minimum and maximum pH of water from *G. truncatula* habitats was given by 29 studies, while the mean pH was given by 8 (or could be calculated from the data provided), of which 1 did not provide the minimum or maximum pH values. The mean minimum pH was 6.1 while the mean maximum was 7.97 and the mean of the means was 7.14 (see Fig. 6). The absolute minimum and maximum pH noted for *G. truncatula* habitats were 5.0 and 9.4.

Of the studies which stated the minimum, maximum and/or mean pH of the habitats where *G. truncatula* was found, 8 provided statistical or stated evidence of the pH preferences in their study, along with 2 other studies which did not provide the pH minimum, maximum or mean pH values. Of these, 3 showed a positive relationship between pH and the snail's abundance or presence, 2 showed a negative relationship and 3 showed no preference or a neutral preference.

### Summary

A summary of the impact of the various environmental factors on *G. truncatula* detailed in the discussion sections below can be found in Table 3.

**Table 3.** Summary of the current understanding of how various environmental factors influence *G. truncatula* and the strength of the effect of these factors

Factor	Association with *Galba truncatula*	Uncertainty of association	Uncertainty of reasons and limits	Notes	Impact on fluke infection and transmission
Identification of the snail	Mixed	Moderate	High	Since 2011, genetic studies have highlighted that morphological identification had frequently misidentified snails as *G. truncatula* instead of other lymnaeid species. As such, there is a level of uncertainty surrounding the life history and geographic range of the snail in general.	Different lymnaeid species have different susceptibilities to infection. Misidentification of the snail species could result in the implication of unnecessary control measures or a misunderstanding of how environmental factors might influence infection rates.
Habitat preference	Mixed	Moderate	High	Rivers/streams (and their banks), drainage watercourses and pools/ponds are the most commonly reported habitats. Differences in habitat terminology and sampling method biases may impact these results, also most common habitats may not be the most optimal. The commonality of streams/rivers does not seem to reflect the snail's preference for bare mud surfaces which are commonly covered in algae, though the snail may have been found on the banks.	Watercourses such as drainage ditches are common on pastureland and could act as the transmission foci for infections. Fencing off these areas from livestock could prevent interactions between the intermediate and primary hosts. However, these sites may be more likely to act as sources of water for livestock rather than grazing, so less common habitats on pastureland may contribute more to fluke transmission as this is where livestock are more likely to consume plants with fluke cysts.
Low Temperatures (Below 10°C)	Strongly Negative	Low	Low	10°C required for reproduction of *G. truncatula*. Some disagreement over the minimum temperature for snail activity, and exposure to low temperatures may be beneficial to juveniles.	Around 10°C is also the minimum reported temperature for fluke development inside the snail, so colder periods are not conducive to fluke transmission.
Mild Temperatures (10 to 25°C)	Slightly Positive	Moderate	High	Most studies suggest the optimum temperatures are within this range, but exact optimum range is unknown. Temperatures in this range at the wrong time of year may cause premature death in juveniles.	As snail abundance is higher at this temperature range, it is likely beneficial for the fluke due to a larger number of intermediate hosts. Targeted Anthelmintic treatments during periods of high snail abundance can be effective at reducing livestock and snail infection rates
High temperatures (above 25°C)	Moderately negative	Low	Moderate	Optimum temperature placed at 25°C by a minority of studies. High temperatures may be detrimental due to low moisture levels.	Likely to be detrimental to the fluke due to decreased intermediate host and water availability.
Water temperature	Moderately positive	Moderate	Moderate	May protect snails from temperature extremes, especially cold air temperatures. However, warmer water temperatures may be beneficial or detrimental.	Potentially can also allow the development of the fluke in areas/times of the year where the air temperature is too cold.
Climate change	Mixed	Moderate	High	Rising temperatures may be beneficial or detrimental depending on local conditions. Extreme weather events and unpredictable weather will make snail presence and infection risk harder to predict.	Warmer temperatures may reduce the number of intermediate hosts available in some areas, though in colder regions it is likely to help spread fluke infection or increase its prevalence.
Rainfall	Mixed	Moderate	High	Moisture needed, but preference for shallow, slow-moving water or moist muddy surfaces could make excess rainfall detrimental. However, flooding may help move snail to new habitats.	High levels of rainfall are commonly associated with high prevalence of *F. hepatica*. Excess rainfall could be detrimental to the snail, but more research needed to assess this.
Altitude	Mixed	Moderate	Moderate	Little impact in temperate climates may have positive correlation in tropical countries, evidence is inconclusive.	Lower infection rates of *G. trucnatula* found in 1 study in France, but high-altitude areas of Bolivia have high primary host infection rates.
Water pH	Limited impact	High	High	Wide pH tolerance range (min 5.0 and max 9.4). Most evidence suggests either a neutral preference or limited impact, but conclusions vary drastically between studies.	Unknown. The optimum pH for the snail is likely to be beneficial for the parasite due to increased availability of intermediate hosts, but this is unknown.
Soil pH	Unknown	High	High	Insufficient data to draw conclusions due to a lack of research.	Insufficient data to draw conclusions due to a lack of research.
Peatland	Strongly negative	Low	Medium	Avoidance of peatland well established, reasons unknown, possibly due to pH or a lack of food.	Presence of peatland may be beneficial for farmers as it could act as a deterrent for snails, thus reducing infection risk.
Dense and clay soils	Moderately positive	Moderate	Moderate	Dense soils with poor drainage are preferred. Some studies show a preference for clay, a dense soil, but others show other soil types.	Areas of farms with poorly draining soils are more likely to pose a risk of infection to livestock, so should be avoided during periods of high snail infection.
Soil organic content	Moderately negative	High	High	Some studies show an aversion to organic content, a possible explanation for peat aversion. Has not been explored in detail.	Not enough detail to determine how this may contribute to fluke transmission.
Salinity and conductivity	Unknown	High	High	Limited number of studies, with a mix of positive and negative correlations observed.	Not enough detail to determine how this may contribute to fluke transmission.
Calcium ion concentration	Slightly positive	Moderate	High	Some studies indicate a positive correlation, 1 paper found a negative correlation.	Not enough detail to determine how this may contribute to fluke transmission.
Habitat permanence	Slightly positive	High	High	Permanent habitats may result in more generations per year, but genetic evidence is unclear over whether population stability is impacted.	If permanent populations are more stable, these may be more likely to act as transmission foci for infection, though this is uncertain.
Soil Disturbance	Mixed	High	High	Soil disturbance often needed to create bare mud surfaces where primary unicellular algae food source grows. But excessive disturbance by animals or vehicles make habitats unsuitable to *G. truncatula.*	The creation of new habitats through soil disturbance could allow for the snails to spread across wider areas of pastureland, which could make controlling infection more difficult.
Independent snail movement	Unknown	High	High	The amount of independent movement *G. truncatula* exhibits is unclear and varies between studies.	If snails are capable of more widespread independent movement, fencing off habitats may be less effective at preventing fluke infection (as the snails could move out of the habitats).
Transport by animals	Strongly positive	Low	High	Transport by animals is reported by several studies. How much the snail distribution is impacted by animals is unclear but could be very impactful.	Could play a major role in fluke transmission, especially if infected snails are transported into previously fluke-free areas.
Transport by flooding	Unknown	High	High	The impact of flooding on *G. truncatula* distribution has not been extensively studied.	Not enough detail to determine how this may contribute to fluke transmission.
Level of shade	Mixed	Moderate	High	Total shade is probably detrimental, but whether fully unshaded or partial shade is more beneficial is unclear.	Farmers are now being encouraged to plant trees, so the placement of vegetation could play a major role in fluke transmission.
Indicator plants	Slightly positive	Low	Moderate	Plants are probably more useful in identifying the general appropriate habitat areas rather than precise habitats. Indicator plant species likely vary between regions.	Not enough detail to determine how this may contribute to fluke transmission.
Drainage systems	Mixed	Moderate	High	Artificial drainage watercourses act as habitats for *G. truncatula*, so may benefit the snail, but draining pastureland may destroy habitats on fields and limit distribution.	Drainage ditches could act as a foci of infection, but could also prevent potential infection across a wider area of field.
Non-agricultural infrastructure	Mixed	Moderate	High	Depending on nature of construction may destroy habitats or allow the snail to spread with livestock along roads.	The destruction of snail habitats may also destroy transmission foci, while fluke may be transported within livestock along roads.
Pollution	Moderately negative	Low	High	Low levels of pollution can be tolerated, but snail prefers lower levels of human activity. Heavy metal pollution can disrupt physiological process.	If pollution negatively impacts *G. truncatula*, infection of livestock is likely to decrease due to fewer intermediate hosts. Unknown whether pollution impacts infection prevalence in the snails.
Habitat restoration and preservation	Unknown	High	High	Schemes to restore and preserve wetland on farmland may create habitats for *G. truncatula*. However, preserving watercourses along the edge of fields may separate *G. truncatula* habitats from livestock. More research is needed to assess potential consequences.	If restored wetland creates new habitats for *G. truncatula*, it is likely to increase infection rates in nearby livestock, but preserving watercourse habitats may separate the transmission foci from livestock.

‘Uncertainty of association’ refers to the level of uncertainty that the factor has the impact most evidence currently suggests. ‘Uncertainty of reasons and limits’ refers to the level of uncertainty regarding the reasons why that factor has those impacts, and the current state of knowledge of the snail's tolerance and preference limits regarding that factor.

## Discussion

### Data comparison

When the results and conclusions of the studies used in this literature review were collated, several issues became apparent which made comparisons more difficult and potentially distorted. The first was that the terminology used often differed between papers and was usually not well-defined. The definitions of the habitats are a prime example of this. Most studies did not define what was meant by each habitat type, and, as such, it was not possible to tell whether 2 studies were using the same parameters to differentiate between habitat types, even if they used the same terminology. Additionally, some studies combined habitat types (such as grouping rivers and brooks together) while others kept them as separate habitats. As pointed out by De Roeck *et al*. (2014) ‘The [small water body] type definitions are not unequivocal in the literature’.

Sometimes it is also difficult to determine where a particular habitat ends, as they are often connected. Peters (1938) discussed how one of the roadside ditches investigated was ‘not merely a ditch to drain the road but was a running stream’. This is especially true when the same water system is sampled at multiple sites, such as the same river or lake, while changes in the water table or recent rainfall could move habitats between types depending on when they were sampled. For this review, different sampling sites were treated as different habitats and so each was counted.

Another potential issue is misidentification. The identification of *Galba* species and the taxonomic relationships of Lymnaeid snails has historically been based on species morphology. However, many of these species are extremely morphologically similar, which makes misidentification a possibility. Bargues *et al*. (2011) revealed that a cryptic species named *Lymnaea schirazensis* (which has since been reclassified as *Galba schirazensis*) had often been confused with *G. truncatula*. Although there is some evidence that *G. schirazensis* is a potential intermediate host of *F. hepatica* (Celi-Erazo *et al*., 2020), at the very least successful infection is much less likely compared with *G. truncatula*, and thus it has likely distorted data related to the susceptibility of *G. truncatula* populations to infection (Bargues *et al*., 2011). Misidentification of snail populations may have also distorted the geographic range of *G. truncatula*, especially as *G. schirazensis* is not the only cryptic species confused with *G. truncatula*. *Galba neotropica* (formerly *Lymnaea neotropica*) also has significant geographic overlap with *G. truncatula* (Bargues *et al*., 2012), while the sub-Saharan populations of *G. truncatula* may be another distinct species (Mahulu *et al*., 2019) and there are also doubts about the populations identified in Alaska and Yukon (Jabbour-Zahab *et al*., 1997).

Due to these cryptic species, any study which only identified *G. truncatula* by morphological characteristics may have misidentified some or all the snails found during their research. However, these studies were still included in the literature review, as this study aimed to assess the overall state of assumed knowledge of the environmental preferences of *G. truncatula*, including the level of uncertainty surrounding these conclusions. With that said, the presence of these cryptic species does mean that any results from morphological-based studies are less reliable, especially in regions where *G. truncatula* is present alongside other morphologically similar lymnaeid species. The bulk of the information in this paper is from Western Europe (specifically France, Switzerland and the UK) where currently there is no record of cryptic species being misidentified as *G. truncatula*, though this is a risk, nonetheless. In most of the rest of this paper, results and conclusions from papers which identified *G. truncatula* morphologically will be compared alongside those that did do using molecular methods as if the species has been correctly identified. However, this is done with the understanding that the morphological identification may not be correct, and thus genetic identification of specimens is needed in future research to validate any findings related to the species environmental preferences. In total, 32/198 studies were confirmed to use genetic identification for every snail used during their research, while 6 only genetically identified a portion of the snails used.

### Habitat preferences

All the *G. truncatula* habitats identified by the studies examined in this review were freshwater sources of some sort, or at least had water present for part of the year. This is not surprising given the snail's physiology. Although *G. truncatula* aestivation can allow the snail to survive for at least 6 months in dry conditions (Kendall, 1949), they will die rapidly unless appropriately sheltered from desiccation, and the egg masses they produce can dry out within 12 h (Roberts, 1950). However, as *G. truncatula* is an air breathing mollusc which must inhale air into its pulmonary cavity to respire, a suitable habitat must allow for regular access to the surface of any water body (Dreyfuss *et al*., 2015). Furthermore, habitats will need to provide a suitable food source, with the snail's preferred natural diet being blue or green algae, which thrives on bare mud surfaces (Euzéby, 1971; Dreyfuss *et al*., 2015).

Given these requirements, it may be somewhat surprising that the habitats found across the largest number of studies were rivers, streams and ponds/pools, as shown in Fig. 2. This may imply that *G. truncatula* is more aquatic than previously thought, however there are a number of alternative explanations. Firstly, several studies included within this literature review were surveys of river systems (Dreyfuss *et al*., 1997; Sîrbu, 2005; Pérez-Quintero, 2013; Phyzhaya and Yanchurevich, 2015; Yigezu *et al*., 2018; Abbaspour *et al*., 2019), and so would obviously include at least 1 river or stream habitat. Secondly, these habitats also included the banks of these watercourses, so given the snail's need for air and the common location of its food sources it seems likely that *G. truncatula* is found on the mud next to rivers and ponds or in the shallower water near the bank rather than in the middle of these habitats. That said, the species was also identified in a diverse range of unique habitats, such as an ‘estavelle-type outlet karst conduit-aquifer’ (Falniowski *et al*., 2021). Though these habitats do not represent the typical water sources that *G. truncatula* inhabits, they nonetheless demonstrate the adaptability of the species.

Overall, habitats which were identified in more studies also tended to be counted more often on an individual basis. This indicates that similar habitats tend to be common for *G. truncatula* across multiple different regions. There are some differences between the 2 counts, however. In some cases, a large number of habitats were found to be inhabited by *G. truncatula*, but were mostly found in only a few studies. For example, Drainage Furrows were the most common habitat in the Individual Habitat count, representing 54.58% of the total habitats counted. However, 10 482 of the 10 626 Drainage Furrows recorded came from just 4 studies, all in central France (Vareille-Morel *et al*., 1999; Rondelaud *et al*., 2011; Dreyfuss *et al*., 2016, 2018*a*).

These regional differences in habitat preferences may be due to different land management causing different habitats to be available, rather than actual regional differences in snail preference. For example, Herzon and Helenius (2008) report that open drainage systems are more commonly found in the Netherlands, Poland and Finland whilst these systems have now almost all been replaced by subsurface piped systems in some countries such as Sweden. Considering the role of *G. truncatula* as an intermediate host for fluke parasites which infect grazing livestock, it is surprising that boggy areas of pastureland were not more commonly recorded as a habitat of *G. truncatula*, as the snail is often found on the surface of damp mud in waterlogged areas. However, this may be partially due to semantics as ‘Swampy/Boggy/Moist Areas’ and ‘Swampy Meadows/Wet Meadows’ may be synonymous, but were kept separate due to differences in terminology and a lack of habitat definitions as discussed in the section ‘Data Comparison’.

It is also worth noting that the most commonly recorded habitats of *G. truncatula* may not be the best suited for the snail, as the 2 habitat counts do not include how many snails were recorded at each habitat. As such, it is possible that some of the more common habitats were only populated by a small number of snails. These data were not collated from studies due to a large variation in defining sampling areas and how the number of snails per habitat was recorded. A few studies did compare the densities of the snail populations in different habitat types, specifically those which investigated the decline in populations in central France (Dreyfuss *et al*., 2016, 2018*b*; Rondelaud *et al*., 2022). These studies seem to suggest that the habitats which are most frequently inhabited by *G. truncatula* tend to have denser populations, though it is unknown if this also applies to other regions. Occupancy can be used as a proxy for species abundance in ecological surveys, including those for which detection is imperfect (Royle *et al*., 2005), but this presupposes that occupancy and abundance are positively and predictably related (Steenweg *et al*., 2018). Fuller characterization of the relationship between observed habitat patch occupancy and abundance of *G. truncatula*, and standardization of methods including calibration of search effort would enable fair comparison between habitats/studies and over time. It is also possible that the presence of *G. truncatula* within habitats is somewhat stochastic or may be driven by factors such as water movement.

Habitat selection in *G. truncatula* may also be driven by the presence of predator species. The terrestrial snail *Zonitoides nitidus* is the most well-studied predator of *G. truncatula*, and natural populations of *Z. nitidus* have prevented the formation of some experimental new colonies of *G. truncatula* though the rate of failure differed substantially between habitat types (Vareille-Morel *et al*., 2002). This species has also been investigated as a potential control mechanism for *G. truncatula*, with *Z. nitidus* shown to eliminate or reduce *G. truncatula* population size within 2 years of introduction. However, new *Z. nitidus* populations were only sustainable in swampy meadows, and *G. truncatula* populations re-established within 3–8 years (Rondelaud *et al*., 2006). Using the omnivorous snail *Oxychilus draparnaudi* alongside *Z. nitidus* resulted in a more rapid elimination of *G. truncatula* populations (Rondelaud *et al*., 2006), and thus, introducing multiple predator species could be a more effective control mechanism.

The larva of sciomyzid flies have also been considered as a potential control method, though this has not been explored experimentally (Gormally, 1985, 1988). To fully assess whether any of these species could be used as a control method, more information is needed regarding their effectiveness and the potential impact their introduction would have on the local ecology of where they were introduced. That said, the use of predators to control mosquito populations has often been effective (Shaalan and Canyon, 2009) though less so in small, artificial habitats (Washburn, 1995).

### Climatic preferences

As shown by Fig. 2, *G. truncatula* has an extensive geographic range across countries with diverse climatic conditions. The full extent of this geographic range is not certain due to misidentification (as discussed in Data Comparison). Nonetheless, the species has been genetically identified in dry climates such as the Bolivian Altiplano (Bargues *et al*., 2021) and North Africa (Mahulu *et al*., 2019), despite the snails need for moisture. This once again reflects the snail's adaptability and ability to colonize suitable habitats within otherwise harsh environments.

*Galba truncatula* can undergo aestivation in order to survive in drought conditions. While this can be an effective strategy in arid climates (Belfaiza *et al*., 2005), many snails die while undergoing this process, and the extent to which it is used seems to vary between populations (Goumghar *et al*., 2001). It would be natural to assume that higher rates of rainfall would therefore be beneficial for *G. truncatula*. However, while some studies have found positive correlations between average rainfall and snail abundance (Walton and Jones, 1926; Charlier *et al*., 2014*a*, 2014*b*), others have found either a negative relationship (Dreyfuss *et al*., 2018*a*) or no significant correlation (Novobilský *et al*., 2014; Roessler *et al*., 2022). These mixed results may be because the species prefers moist mud surfaces or shallow, slow-moving or still water over deep, fast-flowing watercourses (Peters, 1938; Heppleston, 1972; Rondelaud *et al*., 2009; Knubben-Schweizer and Torgerson, 2015; Olkeba *et al*., 2020; Jones *et al*., 2024*a*). As such, too much rainfall could be detrimental to the snails as it could result in too much water being present and/or the water flowing too quickly. This is supported by some studies which have found that *G. truncatula* habitats are less common in areas with both extremely low or high monthly rainfall (De Kock *et al*., 2003), and that the snails are less abundant at times of the year with the lowest and highest rainfall (Relf *et al*., 2011; Iglesias-Piñeiro *et al*., 2016).

In addition to rainfall levels, temperature can also have a major impact on the viability of snail populations. A recent literature review of lymnaeid life history (Dube *et al*., 2023) found that temperatures between 10 and 25°C are generally accepted as optimal for *G. truncatula* fecundity and growth. The findings of this present review are broadly in agreement with this assessment. A minimum temperature of 10°C for at least part of the year is widely accepted as the minimum temperature for a viable *G. truncatula* population as this is the lowest temperature at which the snails produce egg masses (Roberts, 1950; Heppleston, 1972; Valero *et al*., 1998; Charlier *et al*., 2014*a*; Dreyfuss *et al*., 2018*a*). Older laboratory experiments have found that *G. truncatula* can remain active at temperatures as low as 1.5°C (Kendall, 1953), however below 5°C their level of activity (including eating) is severely decreased and this exposure also reduced the number of eggs the adults produce once they are returned to room temperature (Hodasi, 1976). Curiously, this second study also found that juvenile snails exposed to cold temperatures tended to rapidly consume food and experience faster growth post-exposure, resulting in snails that were slightly larger than the control group and produced more eggs. This could indicate that in climates with cold winters, reproduction in the spring is disproportionately driven by overwintering juveniles, although it is not clear whether seasonal exposure to low temperatures increases overall population growth rates.

Overall, though, most research indicates that temperatures below 10°C are suboptimal at best for *G. truncatula*. The impact of higher temperatures, however, is much less certain, as is the optimum temperature for the snail within its tolerance limits. Laboratory tests in the 1950s showed conflicting results, with some data showing that growth rates increased at 25°C compared to 17°C (Roberts, 1950) and other data indicating that 17–21°C is optimal and temperatures above 25°C are detrimental (Kendall, 1953). More modern laboratory studies focused on temperature were found during this review, but they examined the impact on trematode infection and growth in the snail rather than the life history of the snails themselves (Rondelaud *et al*., 2013; Vingnoles *et al*., 2014; Titi *et al*., 2014). As such, these studies were not included in the summary statistics.

Data from field studies are also unclear, partially due to the impact of confounding factors. For instance, most longitudinal studies show that *G. truncatula* abundance does not peak during the hottest months of the year. In some cases, a summer generation occurs shortly before (Charlier *et al*., 2014*a*) or after (Relf *et al*., 2011) the annual peak in temperature, while in other cases no population increase occurs during summer (Mekroud *et al*., 2002; Villavicencio *et al*., 2006; Iglesias-Piñeiro *et al*., 2016). However, the temperatures in which these population peaks occur also differ between studies. In Algeria, the highest snail abundance occurred when mean monthly temperatures reached around 20°C (Mekroud *et al*., 2002), while in Ireland the hottest period still had low snail abundance despite only reaching 15–17°C (Relf *et al*., 2011). Temperature alone is clearly not the core driver of lower population abundance during the hottest parts of the year. Likely it is also due to the increased chance of desiccation due to lower rainfall levels and increased speed of drying out at higher temperatures.

Comparisons of the mean annual temperature of different habitats also have contradictory results. In central France, *G. truncatula* presence was found to have a positive correlation with temperature (Dreyfuss *et al*., 2018*a*), while in South Africa it had a negative correlation (De Kock *et al*., 2003). However, the mean annual temperatures recorded in central France (<9.5 to ⩾11.5°C) straddled the border of the lowest 2 categories temperature categories in South Africa (5–10 and 10–15°C). As such, it is possible that the optimum annual temperature for *G. truncatula* lies around 11–12°C, but it seems more likely that the difference in correlation is due to other factors such as rainfall, habitat availability or soil type.

This could also reflect the preferences of 2 different species, given the uncertainty of *G. truncatula* in Sub-Saharan Africa (Mahulu *et al*., 2019). The discrepancies in temperature preferences between different populations of snails could also be due to the snails adapting to local temperature conditions. Prior literature does seem to suggest differences in reproductive cycles and growth in lymnaeid species adapted to different climates (Dube *et al*., 2023). However, no studies were found which directly compared the temperature tolerances of *G. truncatula* populations from different climates in lab or field observations during the searches for the present review. As such, it is unclear whether any adaptation to different climates is due to evolution or the innate ability of plasticity of the species to upregulate metabolic pathways for thermoregulation.

Temperature can also be significantly altered by local conditions, and so macroclimatic temperature measurements may not be the most accurate indicator of snail presence. Indeed, temperature readings taken closer to the soil may be a more accurate predictor of snail presence, while soil moisture and the presence of plants can also change the intensity of temperature fluctuations (Smith and Wilson, 1980; Roessler *et al*., 2022). Furthermore, water temperature may differ significantly from air temperature, which could help shelter *G. truncatula* from temperature extremes. In the Bolivian altiplano, the average air temperature stays below 10°C for most of the year, while water temperatures mostly stay above this minimum threshold (Bargues *et al*., 2021). Water and shade may also create microclimates that shelter *G. truncatula* from high temperature extremes (Khallaayoune and El Hari, 1991), though without water temperature measurements this is less certain. Some longitudinal studies also found that *G. truncatula* abundance is correlated with periods of increased water temperature (Chlyeh *et al*., 2006; Rondelaud *et al*., 2009; Şahin and Zeybek, 2020), though Olkeba *et al*. (2020) found that water temperature was significantly negatively correlated with *G. truncatula* presence.

Climatic conditions change with altitude, but it is unclear whether this impacts the snail, and outcomes may vary depending on the region. Most studies which focused on altitude in Europe (and 1 in Morocco) found that altitude had little to no impact on the presence of the snail (Walton, 1918; Sturm, 2007, 2012; Georgiev and Hubenov, 2013; Anistratenko *et al*., 2019; Roessler *et al*., 2022). A few studies indicated that altitude may negatively impact the snail, though this may be due to other factors such as higher predation rates or a higher abundance of peatland (Rondelaud and Mage, 1990; Dreyfuss *et al*., 2018*a*). In South America and sub-Saharan Africa, however, several studies identified that *G. truncatula* tends to be found at higher altitudes compared with other lymnaeid species (Walker *et al*., 2008; Pointier *et al*., 2009; Howell *et al*., 2012; Malatji *et al*., 2020; Pereira *et al*., 2020; Bardales-Valdivia *et al*., 2021).

This could indicate that in tropical regions, the temperatures at lower altitudes are too high for *G. truncatula*, but the moderating effect of altitude on temperature allows the snail to thrive. Indeed, the species has been found at lower altitudes in Chile compared to Bolivia, which is closer to the equator (Artigas *et al*., 2011). In South Africa, De Kock *et al*. (2003) found *G. truncatula* across a wide range of altitudes. However, altitude also had a significant impact on the presence of the snail, as 81.6% of collections were between 1000 and 2500 m. Strangely, the lowest altitude areas (0–1000 m) tended to have fewer habitats, but a larger number of snails collected compared with the highest altitude areas (2000–3000 m) in which the trend was reversed. This somewhat mixed result could be a result of South Africa's subtropical to temperate climate.

The varied climatic conditions of different populations of *G. truncatula* also reflect how the species will respond to climate change in different regions. In central France, the species has declined in recent years due to a series of heatwaves (Rondelaud *et al*., 2022; Vignoles *et al*., 2022), while in Bolivia warmer temperatures are allowing *G. truncatula* to colonize areas which were previously too cold (Bargues *et al*., 2020). In the UK, temperatures are staying above 10°C for a larger portion of the year, which is predicted to increase fascioliasis prevalence in livestock (Fox *et al*., 2011). The impact of warmer weather can also depend on the time of year and have a mixed impact. Heppleston (1972) observed that a mild autumn allowed for an additional generation of snails, but this resulted in the premature deaths of many juvenile snails instead of overwintering, while in summer both *G. truncatula* growth and mortality rates increased with higher temperatures.

As climate change makes weather patterns more unpredictable, the observed effects of climate anomalies on snail populations will doubtless depend on what the key limiting factors are in different seasons and locations. When trying to predict the impacts of these weather patterns, it is important to consider the likely curvilinear relationships of *G. truncatula* presence and behaviour with both rainfall and temperature. As increased rainfall and temperature could easily have both a positive and negative impact on snail populations, simple correlations and linear regression, whether univariate or multivariate, might therefore be poorly suited to characterize non-monotonic temperature–abundance relationships.

### Physio-chemical properties of habitats

The impact of differences in the physio-chemical properties of *G. truncatula* habitats on snail presence and abundance has been widely noted but mechanisms are poorly understood. The most widely reported physio-chemical property from *G. truncatula* habitats was water pH. The minimum and maximum pH values recorded demonstrate that *G. truncatula* can tolerate a wide water pH range, while the range of means (6.35–7.8) could indicate that optimum pH range is close to neutral (see Fig. 6). This aligns with research from Europe and South America (Walton and Wright, 1926; Euzéby, 1971; Bargues *et al*., 2021), but this is far from certain given the proportion of studies which identified positive or negative associations between pH and the presence of the snail. Assessing pH preferences is also made more complicated due to potential confounding factors. For example, 1 study found *G. truncatula* in waters below pH 7.4, but lower pH waters in this area were polluted (Pérez-Quintero, 2011), while a study in Wales suggested that the negative correlation between *G. truncatula* and pH could be due to more acidic soils tending to be in higher rainfall areas (Jones *et al*., 2021). Furthermore, Moens (1981) identified *G. truncatula* in habitats with water pH ranging from 5.4 to 8.7 but found the pH level closely aligned with that of the soil in the general region where the habitat was located. This indicates that habitat pH may have little impact on *G. truncatula* presence so long as the pH is within the species' tolerance limits. Only a single study was identified during this review that measured the soil pH of *G. truncatula* habitats (Charlier *et al*., 2014*a*), which found a positive correlation between soil pH and snail abundance, though another study, found after the study searches were completed, found *G. truncatula* at habitats with soils ranging in pH from 4.5 to 6 (Bishop, 1981). This lack of data warrants further investigation into the relationship between soil pH and *G. truncatula* presence.

Regarding soil conditions, several papers noted that *G. truncatula* avoids areas of peatland (Walton, 1918; Peters, 1938; Heppleston, 1972; Moens, 1981; Dreyfuss *et al*., 2018*a*), while only a single paper identified during the review found *G. truncatula* on ‘peaty soils’ (Gaasenbeek *et al*., 1992). It was initially suggested that the assumed avoidance of peatland in *G. truncatula* was because the acid conditions would be detrimental to shell construction (Walton, 1918; Peters, 1938). Lower pH conditions have been found to be detrimental to shell construction in marine gastropods (Duquette *et al*., 2017) and decreased abundance in terrestrial snails (Johannessen and Solhøy, 2001), and the pH can reach as low as 3.8 in some North American peat bogs (Schwintzer and Tomberlin, 1982; Craft, 2022). As such, it is possible that acidic conditions could be one of the reasons for avoidance, particularly in more acidic peatland.

However, the acidity of peatland varies greatly depending on access to groundwater and local minerotrophic conditions, and some areas of peatland can be roughly neutral (Craft, 2022), with these variations potentially impacting suitability for *G. truncatula* inhabitation. For example, a recent study of rich fen peatlands in Finland found *G. truncatula* in 15/19 investigated sites, where water pH was 5.8 on average (Bocharnikova, 2024). Fens, which are fed by mineral-rich groundwater and tend to have a higher pH compared to peat bogs which are mainly fed by rainwater, may therefore be a more suitable habitat for *G. truncatula*. Nevertheless, *G. truncatula* snails were inconsistently detected by Bishop (1981) in East Anglian fens, with *G. truncatula* snails found in fens with a soil pH of 4.5, but not in others where soil pH was as high as 7.6. As such, pH is unlikely to be the sole reason for the avoidance of peatland given the snail's wide pH tolerance.

An alternative hypothesis suggests that the avoidance of peatland may be due to a lack of food caused by an absence of suitable conditions for algal growth (Heppleston, 1972). *Galba truncatula* has reportedly rapidly infested peatland following the application of fertilizer, possibly due to increased unicellular algal growth (Comerton and Houghton, 1975), though both cyanobacteria and chlorophyceae have been identified on peatland (Hamard *et al*., 2021). Whether the species present in these habitats are not favoured by *G. truncatula* or if the algae are not found in sufficient quantities to sustain the snail has not been investigated by any study found during this present review.

The reasons for the apparent aversion to peatland found in *G. truncatula*, and to what extent the snails are averse, needs further investigation. If peaty soils reduce *G. truncatula* numbers and fluke infection, this could act as an incentive for farmers to restore and preserve peatland habitats on their pastures. If *G. truncatula* are averse to peatlands or indeed to general acidic conditions, research would also be needed to establish if other snail species can inhabit these habitats and act as an intermediate host for fluke parasites as has been hypothesized by Relf *et al*. (2009).

The physical properties of the soil may also influence the snail's presence. Several studies have indicated that clay soils are preferred by *G. truncatula* (Walton, 1918; Charlier *et al*., 2014*a*; Dmitrović *et al*., 2016; Vignoles *et al*., 2022). This may be because clay is easier to burrow into compared with other soils (Vignoles *et al*., 2022), as snails have also been found significantly more frequently in habitats with muddy substrata than those that were sandy or stony (De Kock *et al*., 2003). Clay soils may also allow for suitable water source habitats to form more easily, as their low hydraulic conductivity causes them to expand and water to form pools rather than easily draining away (Yu *et al*., 1993; Turtola and Paajanen, 1995). Some studies have found that soils with poor drainage more broadly are positively associated with *G. truncatula* presence, rather than specifically those with a high clay content (Moens, 1981; Wright and Swire, 1984). Additionally, 1 study found that the snail was more common in loamy soils compared to clay soils (Charlier *et al*., 2011), though the reasons for this are unclear.

Roessler *et al*. (2022) also found a negative correlation between *G. trunctula* presence and soil depth (defined as the depth that plant roots can penetrate). This was hypothesized to be due to soil horizons formed from dense particles at a shallow depth preventing root penetration and resulting in poor drainage. Soils with high organic content may also be unfavourable for the snails, as less snail eDNA has been found in darker soils (Jones *et al*., 2024*a*) and *G. truncatula* was found to be significantly less abundant in soils made up of decaying plant matter (De Kock *et al*., 2003). Overall, most evidence suggests poorly draining soils are beneficial for *G. truncatula*, but more research is needed to determine whether the snail favours specific soil types.

Salinity and conductivity may also influence the presence of *G. truncatula*, but the impact of these factors is not well studied and varies between studies. Conductivity was positively associated with *G. truncatula* presence in Austrian lakes and the Normandy Coast (Costil *et al*., 2001; Sturm, 2007), whilst the snail was only found in saline-alkali environments in Northeastern China (Wu *et al*., 2019). However, in Uganda the species had a significant negative correlation with both salinity and conductivity (Howell *et al*., 2012), and in South Africa the species was significantly more likely to be found in freshwater than brackish water (De Kock *et al*., 2003). Research into the impact of concentration of different minerals in the water on *G. truncatula* is also limited. In the First River in Turkey, Calcium was found to have a positive correlation with *G. truncatula* abundance, and was the only mineral studied to have a significant impact on the species' abundance (Şahin and Zeybek, 2020). However, in the rift valley of Ethiopia, the occurrence of *G. truncatula* was positively linked with calcium concentration and negatively linked with magnesium concentration, but the minerals had the opposite impact on the snail's abundance (while nitrate was also shown to positively impact abundance) (Olkeba *et al*., 2020). It is not clear why this is the case, nor why the effects differ between the 2 studies. Overall, more research is needed to clarify the impact of these minerals on snail populations. While farmers may only have a limited ability to change the chemistry of the water on their land, understanding how water chemistry influences the snail will allow for more accurate risk assessments and for planning parasite control measures.

### Habitat permanence, population structure and movement

Some evidence suggests that permanent habitats may be better suited for *G. truncatula*, as populations in temporary habitats are found less often (Moens, 1981; De Kock *et al*., 2003) and tend to have fewer annual generations (Bargues *et al*., 2021). Furthermore, Heppleston (1972) found that populations in permanent habitats have larger populations despite increased mortality, which could indicate that these populations can ‘afford’ more losses due to more optimal conditions. Higher concentrations of snail eDNA have been found in temporary habitats, though this may be due to evaporation concentrating the eDNA rather than an indication of more snails being present (Jones *et al*., 2024*a*).

These suboptimal conditions may be due to the habitats being without moisture for prolonged periods of time, which may cause crashes in the population. In fact, permanent habitats may sometimes act as a reservoir for temporary populations. This was hypothesized to be the case in the Doukkala district of Morocco, in which there were high rates of fascioliasis in livestock despite very few snails being found (Belfaiza *et al*., 2005). It was thought the irrigation cycles might allow snails from a breeding population near a dam to spread through the area, expanding colonized habitats and parasite transmission. These possible cycles of population crashes are reflected in some results which found lower genetic diversity, allelic richness and effective population sizes in temporary populations, which is likely the result of more frequent genetic bottlenecks (Trouvé *et al*., 2003, 2005). That said, other studies have also found a similar level of genetic diversity in populations from temporary or permanent habitats, suggesting no significant difference in the frequency of bottlenecks or population extinction events (Chapuis *et al*., 2007). The same study also found that individuals from temporary habitats mature later and are less fecund (Chapuis *et al*., 2007), which is likely because larger individuals are less susceptible to drought conditions in temporary habitats, while size had no impact on this trait in permanent habitats (Chapuis and Ferdy, 2012).

Heppleston (1972) also observed that snails in temporary habitats exhibited a greater degree of dispersal than those from permanent habitats, though overall the snail was ‘relatively sedentary’, despite earlier laboratory tests indicating the species can move around 33 cm h^−1^. This limited movement is also supported by population genetics which indicate very limited migration between subpopulations (Trouvré *et al*., 2005; Correa *et al*., 2017). However, some field evidence shows *G. truncatula* can move over 100 m per month downstream (Rondelaud, 1983), or 10 m per 2 weeks upstream under favourable conditions (Rondelaud *et al*., 2005). This suggests that *G. truncatula* can move substantial distances, but only do so under certain circumstances such as when exposed to a lack of resources. However, the circumstances which cause the species to move are also poorly understood, so more research would be needed to test this hypothesis.

Questions also remain regarding the potential for *G. truncatula* to be transported by animals either locally or across vast geographical areas. Juhász and Majoros (2023) found that wild boars transported *G. truncatula* from wallowing sites on their coat whilst it has been hypothesized that Equids and Llamas may carry and transport the snail on their feet (Mas-Coma *et al*., 2020, 2021; Mera Y Sierra *et al*., 2020). As such, it is also feasible that livestock may transport the snail on their hooves, which could be an important means for *G. truncatula* to colonize temporary habitats on pastureland, however, no evidence of such a phenomenon was found in the literature. The ability of *G. truncatula* to undergo aestivation in dry mud may also allow long distance transport on the feet of livestock, with Mas-Coma *et al*. (2009) hypothesizing that it was by this means that *G. truncatula* initially arrived in South America as cattle were imported to the continent from Europe from the 1400s onwards. It has also been suggested that birds can aerially distribute *G. truncatula* over thousands of kilometres and allow the snail to colonize remote areas such as mountains and islands, with the global distribution of *G. truncatula* sharing similarities with major migratory flightpaths of wetland birds (van Leeuwen, 2012). The mode of such transport is unclear though, with transport attached to the feet or feathers most likely, although some molluscs are capable of surviving passage through the avian digestive tract (van Leeuwen, 2012).

Snails may also be dispersed by flowing water along waterways. Hurtrez-Boussès *et al*. (2010) found that genetic differentiation of snail populations increases with hydrographic distance along the streams, and the genetic variance between populations is lower than expected if the populations were connected by direct water flow. This could be an important consideration for combatting the spread of *G. truncatula* and fluke infection.

The influence of flooding on the spread of the *G. truncatula* is unclear. Although flooding could spread the snail a considerable distance from permanent habitats (Walton, 1918), some researchers suggest that most of the habitats the snails are placed in are likely to be unsuitable (Peters, 1938), and that flooding is unlikely to be the main contributor of snail dispersal into new regions (Heppleston, 1972). However, flooding may also transport some *G. truncatula* into new suitable habitats where they can rapidly establish new colonies (Skuce *et al*., 2014). This may be especially true in the period immediately after flooding, following the creation of many temporary habitats on pastureland away from the snails' typical habitats. These temporary habitats may also last for several weeks if the pasture has poor drainage or if rainfall continues after the flooding subsides. As such, while the heavy rainfall that results in flooding could be detrimental to the snails in permanent habitats (as discussed above in ‘Climatic Preferences’), it may also allow for either the temporary or permanent expansion of the snail's range, and thus the spread of trematode infections.

There is limited research focused on the vertical movement of the snail. Research focused on a population downstream of a dam found that snails mostly followed seasonal variation of water levels, but more rarely followed daily fluctuations. However, some individuals did retreat to emerged ground when water levels rapidly increased (Hourdin *et al*., 2006). Preference for water depth may differ between populations, with snails from marshlands appearing to be more amphibious than those from populations in irrigation and road ditches, while infection by *F. hepatica* resulted in a preference for deeper water (Moukrim and Rondelaud, 1992). Overall, how *G. truncatula* moves in response to changes in water depth is poorly understood.

### Radiation, shade and habitat flora

There is some evidence that high levels of shade may be detrimental for *G. truncatula*, although it is unclear whether some shade is beneficial. Most studies suggest that bare mud surfaces are preferential for *G. truncatula*, as these are good conditions for the unicellular algae on which the snail feeds (Kendall, 1953; Heppleston, 1972). However, evidence from Spain found that *G. truncatula* is positively associated with daily global radiation up to 18 000 kJ (10 kJ m^−2^ day^−1^), but then declines (Iglesias-Piñeiro *et al*., 2016). This may suggest that some shade is beneficial, which aligns with findings which showed *G. truncatula* presence is significantly less likely for habitats located within forests but significantly more likely if they are near to forests (Roessler *et al*., 2022). Furthermore, a lack of vegetation along riverbanks was hypothesized to be the reason for smaller riverbank populations of *G. truncatula* compared to marshland populations (Dreyfuss *et al*., 1997), while De Kock *et al*. (2003) also found that aquatic vegetation was present at 91.6% of *G. truncatula* habitats (though it was only calculated to have a small impact on the likelihood of the snail's presence).

However, Jones *et al*. (2024a) found that while there was significantly lower levels of *G. truncatula* eDNA in fully shaded habitats, no significant difference was found between partially shaded and unshaded habitats, which indicates the benefits of partial shade may be more situational. This study also highlighted that farmers in many parts of the world are being encouraged to plant trees for environmental purposes. As such, understanding whether the shade provided by canopy vegetation is beneficial or detrimental to the snail could be important for farmers to strategically place trees to interrupt fluke transmission and prevent livestock infection. Careful siting of trees could also increase hydraulic connectivity and reduce soil saturation in areas of pasture acting as foci of snail populations.

Plants are also sometimes used to identify potential habitats of *G. truncatula*, although studies differ as to whether specific plants are associated with the presence of the snail or not. For example, rushes (family Juncaceae) have been associated with the presence of *G. truncatula* in a number of studies (Rondelaud *et al*., 2011; Dreyfuss *et al*., 2018*a*; Jones *et al*., 2021, 2024a), but Charlier *et al*. (2014*a*) found no significant correlation between the presence of rushes and *G. truncatula*. This study also found a negative correlation between the presence of *G. truncatula* and reeds, but a more recent study found a positive correlation (Roessler *et al*., 2022). Buttercups (genus *Ranunculus*) are another potential indicator, as they were commonly found at *G. truncatula* habitats by Charlier *et al*. (2014*a*) and Roberts, (1950). It seems likely that botanical indicators of *G. truncatula* presence may vary substantially geographically and between habitat types.

Three of the studies in this review identified the presence of *G. truncatula* in watercress beds (Rondelaud and Mage, 1990; Rondelaud *et al*., 2005, 2006). Though this indicates this is an uncommon habitat of the snail, it is troubling as *Nasturtium officinale* (common watercress) is the main cause of human infection by *F. hepatica* in Western Europe (Rondelaud *et al*., 2005). As such, it is important that the crop is carefully monitored for the snail, and water supply regulated especially to avoid ingress of fluke eggs.

Snails from different habitats may also be adapted to consume different types of plant matter and algae based on the availability in their environment. Vignoles *et al*. (2011) reared snails from a riverbank population on conditions optimal for those from meadows but found no significant increase in snail size compared with those in the wild habitats. It was suggested that the decaying leaves used as feed in meadows may not make up a large part of the river snails' diet, and that they might therefore have developed different food preferences. A recent metagenomic study of the gut microbiome of snails from 2 farms revealed significantly different beta diversity, which indicates that the microbiome may be adapted to different conditions (McCann *et al*., 2024). This could possibly be due to differences in the snail's diet, however the diet of these 2 populations is not known. Metagenomic studies of the gut contents of snail intermediate hosts are rare, and no genetic studies of *G. truncatula* diet were found during this review.

### The impact of human activity

The creation and destruction of snail habitats are perhaps the most direct impacts that humans can have on the presence or abundance of *G. truncatula*. However, whether a certain human activity is beneficial or detrimental to the snail seems to be very situational. Artificial watercourses such as drainage ditches, drainage furrows and irrigation canals are among the most abundant habitats of *G. truncatula* across many different studies (Rondelaud *et al*., 2011; Charlier *et al*., 2014*a*, Dreyfuss *et al*., 2016, 2018*a*, 2018*b*; Vignoles *et al*., 2022; Jones *et al*., 2024*a*). This may be because these artificial watercourses are similar to natural habitats such as streams and rivers, and thus the creation of these habitats may allow for the presence of the snail. However, in many cases these artificial habitats are part of open drainage systems and draining fields can be an effective way to destroy *G. truncatula* habitats (Rees, 1942; Dreysuss et al., 2016, 2018*a*, 2018*b*). Furthermore, when subsurface drainage systems malfunction, they can create new habitats through subsurface effluences which can potentially hamper efforts to combat Fascioliasis (Bargues *et al*., 2020; Jones *et al*., 2024*a*). As such, open drainage systems may act as an effective habitat for *G. truncatula* while simultaneously limiting their spread across the field by concentrating the moist habitats in 1 area. That said, large-scale interconnected artificial waterways may also allow for *G. truncatula* and fascioliasis to spread, as was found by Belfaiza *et al*. (2005). Regular clearing of open drainage systems could be an effective method of control, as it is one of the main reasons for habitat loss of *G. truncatula* in central France (Dreyfuss *et al*., 2016, 2018*b*).

*Galba truncatula* can also be impacted by poaching (the mechanical disturbance of soil and vegetation in wet conditions) by vehicles or livestock. Tracks created by vehicles and livestock can fill with water and thus act as habitats for the snail (Heppleston, 1972; Hörweg *et al*., 2011; Dreyfuss *et al*., 2016; Jones *et al*., 2024*a*). However, excessive poaching may also be detrimental for the snail. Charlier *et al*. (2011) found no *G. truncatula* in humid areas near water troughs, while Dreyfuss *et al*. (2018*b*) found that cattle trampling has resulted in the destruction of *G. truncatula* habitats near vernal pools used as a water source. It is possible that the high throughput of animal traffic in these areas results in the mechanical disturbance of snail habitats, though they are also likely to be subjected to a high degree of urine and faecal deposition, a source of pollution which will increase habitat microbial load (O'Callaghan *et al*., 2019) which is likely to be detrimental to *G. truncatula*. High animal or vehicle traffic may also result in the destruction of the snail itself, as Juhász and Majoros (2023) noted that while vehicles can decrease soil drainage (which can be beneficial for *G. truncatula*), they can also crush the snails beneath their tires.

Mechanical gyro-crushing of rushes and other vegetation near small water sources has resulted in a decline of *G. truncatula* habitats in central France (Dreyfuss *et al*., 2016, 2018*b*), possibly because the deposition of crushed plant matter on the mud surface inhibits unicellular algal growth (Dreyfuss *et al*., 2018*a*, 2018*b*). It is unknown if the destruction of rushes by other means such as topping would have a similar effect, though the practice is regularly promoted as a method of fluke control (COWS, 2019). Mowing has also been found to correlate negatively with the presence of *G. truncatula* (Charlier *et al*., 2014*a*), which may be due to the intense regeneration of plants after mowing hindering the production of the unicellular algae (Dreyfuss *et al*., 2016).

Non-agricultural infrastructure can also impact the presence of the snail. In Bolivia, road construction disrupted fascioliasis transmission loci, but allowed the snails to be transported to new areas by livestock carried on trucks (Bargues *et al*., 2020). Additionally, unpublished data by Rondelaud (reported by Hourdin *et al*., 2006) shows that river habitats upstream of dams tend to be numerous with high snail population densities. Several studies have also shown that while *G. truncatula* can tolerate low levels of pollution, the species tends to prefer areas with less anthropogenic activity (Pérez-Quintero, 2011; Phyzhaya and Yanchurevich, 2015; Bargues *et al*., 2021). However, this may depend on the activity as Olkeba *et al*. (2020) found that car-washing, and silviculture had a positive correlation with the presence of the snail. Heavy metal pollution in Iran has been found to supress antioxidants, neurotransmitter activity and compounds used for protein synthesis (Banaee and Taheri, 2019), while pesticide pollution in the same river also led to decreased antioxidant activity in the snail (Raisi *et al*., 2018).

Global climate change may be having a significant impact on the distribution of *G. truncatula*, though whether this impact is positive or negative largely depends on the region in question. However, in recent decades policymakers have encouraged farmers to preserve or create areas of biodiversity such as wetlands and woodland to preserve species and mitigate global warming (Brown, 2021; Sidemo-Holm et al., 2021; Collas, 2023). The potential impact of woodland due to shade is discussed above (Jones *et al*., 2024*a*). Creating new wetlands could introduce new habitats and therefore high-risk areas for livestock infections by trematodes. For example, research by Cuthill (2020) showed that scrapes, which are small, shallow, irregular-shaped pools created on pasture as habitats for declining farmland wading birds can be rapidly colonized by *G. truncatula*.

As such, concerns have been raised regarding the impact of widespread farmland scrape creation on liver fluke infection risk in livestock (Jones *et al*., 2024*b*). The risk of scrapes becoming a source for *Fasciola* infection will likely vary between farms depending on the precise location of the scrape, the environmental properties of that location and how the scrape is grazed and how the wider area is managed. However, restrictions are often placed upon the management of wetland habitats of biodiversity value. As such practices often promoted to limit fasciolosis risk including fencing, rotational grazing and drainage cannot be implemented on some wetland habitats where *G. truncatula* populations exist as strict conservation grazing and land management regimens are a necessity to maximize biodiversity (Martin *et al*., 2013). But even in instances where new wetland habitats are fenced off, they may still impact fluke risk primarily through its hydrological effect on neighbouring grassland.

Restoring wetland habitats such as fen and peat bog near pastureland could attenuate any potential positive effects on *G. truncatula* due to its aversion to peat. Substantially more research is needed to assess the impacts of both afforestation and peat/wetland restoration on *G. truncatula* ecology and liver fluke risk, with very few longitudinal or intervention studies discovered in the present review. There are also many socio-economic dimensions of ecological restoration processes, with arguments for and against their integration with food production (Brown, 2021; Collas, 2023), but animal health is not usually taken into consideration. Additionally, due to the increasing demand for livestock produce, there have also been several studies investigating the viability of alternative crops for foraging (e.g. Kakabouki *et al*., 2014; Lee *et al*., 2021; Karydogianni *et al*., 2022). It is unknown whether using these plants instead of traditional grasses would impact the snail's ecology.

The cost of land modification strategies must also be considered in addition to their effectiveness at preventing fluke infection. Improvements to drainage systems, changing foliage and so on may be very expensive, and thus divert resources from other investments. This will be exacerbated in lower income regions and countries, which are also more likely to have substandard sanitation infrastructure which may undermine attempted control measures and contribute to the spread of fascioliasis to humans. In the Bolivian settlement of Huacullani, children were found to be at risk because the route they took from school crossed transmission hotspots. Similar problems have impacted the control of mosquitoes in Ecuador, where a lack of sanitation has led people to store water in ways that encourage mosquito proliferation (Harris and Carter, 2019). As such, any control measures developed will need to take local financial and infrastructure conditions into account to be successful.

### Ecology and fluke transmission

Environmental conditions which impact populations of *G. truncatula* may also influence cycles of fluke transmission. Several studies have shown that fluke infection rates vary between habitats, with lower rates identified in river and stream populations (Schweizer *et al*., 2007; Rondelaud *et al*., 2016a) and in snails that inhabit habitats with fast-flowing water (Manga-Gonzalez *et al*., 1991). This may be due to the faster flowing water being sub-optimal for the snail or impeding the ability of the miracidia to swim to snails. Additionally, the diversity of trematode species infecting *G. truncatula* can also vary depending on habitat type. Rondelaud *et al*. (2016*a*) found that only *C. daubneyi* and *F. hepatica* were found in *G. truncatula* from drainage furrows, while there was a much greater diversity in pools. Furthermore, *Opisthoglyphe ranae* and *Haplometra cylindracea*, trematode parasites of frogs, had much higher infection rates in pools than *F. hepatica*. It is unknown whether this is due to differing habitat preferences of the trematodes or the definitive host species being more likely to frequent different habitats. Although the intensity of co-infection of either *F. hepatica* and *C. daubyeni* in livestock has been found to be negatively correlated with the other species (Jones *et al*., 2017*a*), it is currently unclear whether habitats that encourage greater competition of trematode species have a reduced risk of transmission to definitive hosts.

Some studies have also identified a positive correlation between snail size and *F. hepatica* infection rates, both within (Smith, 1984; Wislon and Denison, 1980) and between snail populations (Chapuis, 2009). However, while there is some evidence that snails in temporary habitats and those from higher altitudes tend to be larger in size (Goumghar *et al*., 2001; Chapuis *et al*., 2007), it is unknown if these populations are more susceptible to infection. Furthermore, in Galicia, Spain, it was found that while snail size was positively associated with *F. hepatica* infection, *C. daubneyi* had a negative correlation, which may be due to older snails developing resistance to *C. daubneyi* over time (Iglesias-Piñeiro *et al*., 2016).

Much like the population abundance of *G. truncatula*, fluke infection rates in the snail also undergo seasonal fluctuations which can vary from year to year due to climatic conditions. However, local conditions can also alter the impact of these weather patterns, as Novobilský *et al*. (2014) found that seasonal variation in the proportion of infected snails differed substantially between farms in Sweden. Several studies have identified that local factors can impact infection rates in livestock (Bennema *et al*., 2011; Charlier *et al*., 2011; Jones *et al*., 2017*a*), however it is difficult to assess whether these factors have a bigger impact on the snails or the trematodes, and in some cases both may be impacted. For example, while mowing may be detrimental for *G. truncatula* (as discussed in ‘The Impact of Human Activity’), it may also remove metacercariae from fields and kill the free-living stages by exposing them to sunlight. That said, the infection rates within the definitive and intermediate hosts are linked. For example, Jones *et al*. (2017*b*) found that the *F. hepatica* and *C. daubneyi* egg count in livestock faeces was the main determining factor of infection rates in *G. truncatula*.

That said, even snail populations with infection rates of <1 to 7% in *G. truncatula* are enough to cause livestock infection rates of over 30% (Khallaayoune and El Hari, 1991; Mage *et al*., 2002; Belfaiza *et al*., 2005). A similar pattern is also seen in human schistosomiasis infection rates (Léger *et al*., 2020; Nwoko *et al*., 2022), and highlights the need to not only identify the habitats where *G. truncatula* is present, but also where the snail is likely to interface with livestock. Currently, there is disagreement over whether *F. hepatica* becomes better adapted to infect local populations of *G. truncatula* over time (Vareille-Morel *et al*., 2002; Dreyfuss *et al*., 2012; Sanabria *et al*., 2013) or is better adapted to infect new populations of snail (Gasnier *et al*., 2000; Correa *et al*., 2017). In either case, preventing livestock from interfacing with the snail will work to both break current cycles of infection and prevent flukes from establishing themselves in new snail populations.

Identifying which potential habitats contain *G. truncatula* and how far they extend across pastureland can be a time-consuming process, and sometimes the snails may be missed during malacological searches due to their small size or low populations. However, eDNA techniques have been proven highly effective at identifying the presence of *G. truncatula*, as well as other amphibious trematode intermediate snail hosts, even in habitats where the snails were not visually identified (Jones *et al*., 2018, 2021, 2022; Davis *et al*., 2020; Mulero *et al*., 2021; Rathinasamy *et al*., 2021). This could be especially useful in identifying habitats which may contribute to the spread of fluke to livestock but are only inhabited by a small number of snails. That said, it is well established that other snail species can act as the intermediate host of *F. hepatica* and *C. daubneyi*, but it is unknown how much they contribute to the spread of these trematodes, especially given the issues with misidentification discussed in ‘Data Comparison’. More research also is needed to investigate the role of these other species in fluke transmission, particularly in environments where *G. truncatula* is not present.

### Environmental management of snails to reduce fluke transmission

It is currently unclear whether the most common or suitable habitats of *G. truncatula* act as the primary transmission foci for fluke infection. Rivers and drainage furrows are the most common according to the habitat counts, but these would primarily be water sources on pastures, rather than a significant source of grazed forage. As such, it may be the case that rarer habitats recorded in this review, such as moist areas or subsoil effluences on the pastureland, contribute more to infection than those watercourse habitats on the periphery of fields as grazing livestock would consume plant matter contaminated with fluke cysts. In this instance, *G. truncatula* populations in these habitats would be worthy of increased focus in preventative control strategies, but this has not been systematically investigated. Farmers are often encouraged to fence off ‘wet areas’ to avoid fluke infection, but large swathes of pastureland may need to be temporarily fenced off to match the same impact as current treatment-based measures. This is especially true in flatter regions where potential habitats are likely to be more widespread (Beltrame *et al*., 2021).

If watercourses are the primary transmission foci for infection, this could be used as an incentive for farmers to take part in schemes to preserve watercourse ecosystems. Arnott *et al*. (2019) found that while funding was available for such projects under Agri-environment schemes, they only accounted for only 3% of options taken up by farmers. However, this incentive relies on whether restricting access to watercourses reduces fluke transmission to livestock. An alternative to fencing is improving better drainage in fields, as Beltrame *et al*. (2021) found that around 8% more drainage on UK farms could match the impact of treatment regimens. However, this strategy runs counter to the need to increase water storage on farmland to alleviate downstream flooding from increasingly frequent extreme rainfall events (Collentine and Futter, 2018).

If environmental measures are not a viable or affordable solution, improved treatment regimens may be a better option in the short term, though this does risk the development and spread of Anthelmintic resistance through overtreatment (Vineer *et al*., 2020). However, targeted treatment regimens could be used to reduce the transmission of fluke eggs onto pastureland at the times of the year when the snails are most active. Such regimens have been shown to not only reduce infection rates of *F. hepatica* in livestock, but also in *G. truncatula* (Parr and Gray, 2000), however, there remains potential for wildlife such as rabbits and hares to also contaminate *G. truncatula* habitats with *F. hepatica* eggs (Juhasz *et al*., 2023).

Alternatively, grazing schedules could be created so that livestock avoid fields with high-risk areas at times when infective metacercariae are most abundant. Livestock are also known to spend more time near water sources for drinking and grazing during periods of dry and warm weather (Boray, 2007; Pandey *et al*., 2009), so providing alternative water sources during these periods could lower the risk of fluke infection.

However, this strategy requires a better understanding of which factors result in high-risk areas so they can be mapped at the farm level, as has been done for other helminth livestock parasites (McFarland *et al*., 2022). Incorporating the preference of *G. truncatula* for poorly draining soils has already been shown to improve the HELF model of fluke risk (Beltrame *et al*., 2018), and earlier research has suggested that mapping potential *G. truncatula* habitats will increase the accuracy of infection risk models (Charlier *et al*., 2011). It stands to reason that gaining a greater understanding of the environmental preferences of *G. truncatula* will allow for a more accurate mapping of potential risk areas. However, which habitats are high risk may also depend on the livestock species, as cattle are known to favour marshier areas of pastureland than those favoured by sheep or goats (Boray, 2007).

Watercress beds were the only habitats investigated by studies in this review in which direct plant consumption could cause human fascioliasis. However, the consumption of freshwater plants is also a major source of infection in the Bolivian altiplano, which suffers from hyperendemic human infection (Angles *et al*., 2022). In both cases, thorough cleaning of the crops is required to ensure cysts are not ingested. However, a lack of treated water available for rural households is also a major issue for regions with hyperendemic human infection, especially as poor sanitation can result in sewage, natural water sources, pasture drainage and irrigation ditches for crops being connected, resulting in the spread of *F. hepatica* from *G. truncatula* habitats to human habitation (Angles *et al*., 2022). As such, improvements to sanitation systems and the education of local community leaders are vital to help reduce human infection rates in these areas.

## Conclusions and future research

The evidence presented in this current review shows that *G. truncatula* is an extremely adaptable species which has managed to colonize a wide range of habitats across a range of climatic conditions. However, many of the environmental preferences of *G. truncatula* are still poorly understood. In some cases, the more general preferences are reasonably well established, but lack precision, such as the optimum depth of water favoured by the snail. In other cases, the absence of research or conflicting information means that very little information is known about the optimum conditions for the snails, such as the impact of water conductivity. Even in the case of the snail's aversion to peatland, the underlying mechanics are not well understood, and recent evidence suggests this well-established facet of *G. truncatula* may be more nuanced than initially thought.

Another potential reason for the lack of clarity may be the impact of confounding factors. Many environmental factors interact with one another, and so it is difficult to assess which is having the larger impact on *G. truncatula* presence or abundance in a particular region or context. This may explain why the impact of certain variables seem to differ between studies, as the impact of a factor such as pH may be confounded by rainfall levels or soil type and so forth. As discussed in the ‘Data Comparison’ section, a lack of standardization between studies also makes comparing data between studies difficult and thus the patterns of environmental preferences of *G. truncatula* harder to assess.

Given these issues, the following recommendations are made for future research. Firstly, a greater number of laboratory studies are needed which explicitly focus on how environmental factors influence the life history of *G. truncatula*. Lab-reared populations of *G. truncatula* have been used in many different studies, primarily used to produce flukes or measure the impact of environmental variables on fluke development. Similar studies could also measure the impact of environmental variables on the snail in a controlled environment to mitigate the impact of confounding factors. This could also be combined with transcriptomics to give a baseline of which cells and genes are active under certain conditions.

Secondly, greater clarity on the methods for identification of snail species and habitat definitions is needed in field studies. Given the issues with morphological misidentification, all collected snails should be genetically identified as *G. truncatula*. This, along with stating how each habitat type was defined, will make inter-study comparisons easier and more reliable.

Thirdly, a greater emphasis is needed on the interactions between environmental factors using multivariate analysis. Longitudinal studies will also allow researchers to account for the impact of seasonal weather changes on snail populations and to compare the effects of years with differing weather conditions. This will become increasingly important as climate change makes seasonal weather patterns more erratic, so measuring the impact of extreme ‘unseasonal’ weather on snails in different areas will be vital in understanding how they affect the spread of trematode infections. Longitudinal studies can also be used to measure the impact of preventative strategies over time and develop new strategies which consider the impact of extreme weather events.

Collaborative research projects using similar methodologies across multiple regions or countries could also be extremely useful. This would allow for data from a wide range of different environments to be compared, and thus differences between distinct populations could be more easily identified. This is especially true if snails from different environments were subject to the same laboratory conditions. While such a project would likely to be time consuming, the use of eDNA technology could help reduce the time and resources needed to identify the snail's presence.

In addition to purely focusing on the environmental preferences of *G. truncatula*, more research is also needed to understand how these preferences influence fluke transmission, along with the role of other potential intermediate snail hosts. Furthermore, the impact of land and livestock management strategies on *G. truncatula* abundance and subsequent fluke transmission need further investigation. Studies of interventions against *G. truncatula* populations should be properly designed using before-and-after or ideally case–control methodologies, so that interpretations are clear and repeatable. These control strategies can be time-consuming and expensive for farmers, and so intervention studies should therefore span a suitable period to show sustained effects. Population models can have a useful role in study design by selecting the interventions most likely to succeed and estimating the effort required for the desired outcome. Although current knowledge of the bionomics of *G. truncatula* does not permit precise predictions, more effort to develop and parameterize such models might be well rewarded.

Ultimately, the goal of investigating the ecology of *G. truncatula* is to better understand the risk factors which lead to the spread of Fascioliasis and other trematode infections in livestock, humans and wildlife. Currently it is still unclear which factors are most important, and how these factors interact. If these are better understood, it will allow for risk assessment models to become more precise and accurate and allow risk areas to be mapped at a farm or even field specific level. This will assist both policymakers and farmers to create more targeted strategies to prevent the spread of infection. It will also allow for a better prediction of the potential impacts of climate change on the spread of these diseases, and thus the creation of evidence-based plans to mitigate their impacts.

## Supporting information

Smith et al. supplementary material 1 Smith et al. supplementary material

Smith et al. supplementary material 2 Smith et al. supplementary material

Smith et al. supplementary material 3 Smith et al. supplementary material

## Data Availability

Supporting data is found within the paper, or from the original papers referenced as part of the systematic literature review. A full list of papers used can be found in the supplementary materials.

## Supplementary material

The supplementary material for this article can be found at https://doi.org/10.1017/S0031182024000957
