# Supplementary material for: Environmental influences on the distribution and ecology of the fluke intermediate host Galba truncatula: a systematic review
Source: Parasitology. 2024 Dec 3;151(11):1201–24. doi: 10.1017/S0031182024000957 (PMC11894016; doi:10.1017/S0031182024000957)
Supplement: Smith et al. supplementary material 2 — Smith et al. supplementary material [file S0031182024000957sup002.docx]

**Supplementary Materials Document 2**

**Risk of Bias and Full List of Included Studies**

**Table S3:** Risk of Bias Assessment using the MMAT (Mixed Methods Appraisal Tool), divided into sections based on categories of study as defined by MMAT guidelines. None of the studies evaluated were Qualitative studies, so section 1 has been skipped.

| **Quantitative Randomized Controlled Trials** | **Screening Questions** | | **Methodological Quality Criteria** | | | | |
| --- | --- | --- | --- | --- | --- | --- | --- |
|  |  | |  | | | | |
| **Study Name** | **S1: Are there clear research questions?** | **S2: Do the collected data allow to address the research questions?** | **2.1** | **2.2** | **2.3** | **2.4** | **2.5** |
| A strategic dosing scheme for the control of fasciolosis in cattle and sheep in Ireland | Yes | Yes | Yes | Yes | Yes | No | Yes |
| A survey of the Lymnaea truncatula habitat areas in Co Down, Northern Ireland | Yes | Yes | Yes | Yes | Yes | No | Yes |
| A survey on the Lymnaea truncatula habitat areas in Co. Tyrone, and Co. Fermanagh, Northern Ireland. | Yes | Yes | Yes | Yes | Yes | No | Yes |
| Diel activity cycles of freshwater gastropods under natural light: Patterns and ecological implications | Yes | Yes | Yes | Yes | Yes | Can't tell | Yes |
| Effects of Pirimicarb carbamate insecticide alone and in combination with lead (Pb) on biochemical parameters of soft tissues in freshwater snail, Galba truncatula | Yes | Yes | Can't tell | Yes | Yes | Can't tell | Yes |
| Evolutionary implications of a high selfing rate in the freshwater snail Lymnaea truncatula. | Yes | Yes | Yes | Yes | Yes | Yes | Yes |
| Field and experimental evidence of preferential selfing in the freshwater mollusc Lymnaea truncatula (Gastropoda, Pulmonata) | Yes | Yes | Yes | Yes | Yes | Can't tell | Yes |
| Relationship between individual metabolic rate and patch departure behaviour: evidence from aquatic gastropods | Yes | Yes | Yes | Yes | Yes | Can't tell | Yes |
| Sub-lethal effects of dimethoate alone and in combination with cadmium on biochemical parameters in freshwater snail, Galba truncatula | Yes | Yes | Yes | Yes | Yes | Can't tell | Yes |
| **Quantitative Nonrandomized** | **Screening Questions** | | **Methodological Quality Criteria** | | | | |
| **Study Name** | **S1: Are there clear research questions?** | **S2: Do the collected data allow to address the research questions?** | **3.1** | **3.2** | **3.3** | **3.4** | **3.5** |
| A new method for laboratory rearing of Galba truncatula, the intermediate host of Fasciola hepatica | Yes | Yes | Yes | Yes | Yes | Yes | Yes |
| A retrospective study on the metacercarial production of Fasciola hepatica from experimentally infected Galba truncatula in central France. | Yes | Yes | Yes | Yes | Yes | Yes | Yes |
| Allopatric combination of Fasciola hepatica and Lymnaea truncatula is more efficient than sympatric ones. | Yes | Yes | Yes | Yes | Yes | Yes | Yes |
| Aplexa hypnorum (Gastropoda: Physidae) exerts competition on two lymnaeid species in periodically dried ditches | Yes | Yes | Yes | Yes | Yes | Yes | Yes |
| Bionomics of Limnaea truncatula and the Parthenitae of Fasciola hepatica under Drought Conditions | Yes | Yes | Yes | Yes | Yes | Yes | Yes |
| Characteristics of Fasciola hepatica infections in Galba truncatula originating from riverbank populations | Yes | Yes | Yes | Yes | Yes | Yes | Yes |
| Comparative strategies and success of sympatric and allopatric Fasciola hepatica infecting Galba truncatula of different susceptibilities | Yes | Yes | Yes | Yes | Yes | Yes | Yes |
| Consequences of invasion by Pseudosuccinea columella on the dynamics of native lymnaeids living on the acid soils of central France | Yes | Yes | Yes | Yes | Yes | Yes | Yes |
| Correlation between parasite prevalence and adult size in a trematode-mollusc system: evidence for evolutionary gigantism in the freshwater snail Galba truncatula? | Yes | Yes | Yes | Yes | Yes | Yes | Can't Tell |
| Detection of Galba truncatula, Fasciola hepatica and Calicophoron daubneyi environmental DNA within water sources on pasture land, a future tool for fluke control? | Yes | Yes | Yes | Yes | Yes | Yes | Yes |
| Experimental colonization of new habitats by Galba truncatula O.F. Muller (Gastropoda : Lymnaeidae) in central France and their susceptibility to experimental infection with the trematode Fasciola hepatica L. | Yes | Yes | Yes | Yes | Yes | Yes | Yes |
| Fasciola hepatica and lymnaeld snails occurring at very high altitude in South America | Yes | Yes | Yes | Yes | Yes | Yes | Yes |
| Galba truncatula (Mollusca Gastropoda, Lymnaeidae), the snail host of Fasciola hepatica: Its ability to withstand a periodic drying of its habitat in an irrigated area tinder a semiarid climate | Yes | Yes | Yes | Yes | Yes | Yes | Yes |
| Galba truncatula (OF Muller, 1774) (Gastropoda, Lymnaeidae): the colonization of new stations on acid soil by low numbers of snails | Yes | Yes | Yes | Yes | Yes | Yes | Yes |
| Growth rate of the intermediate snail host Galba truncatula influences redial development of the trematode Fascioloides magna | Yes | Yes | Yes | Yes | Yes | Yes | Yes |
| Highland populations of Lymnaea truncatula infected with Fasciola hepatica survive longer under experimental conditions than lowland ones | Yes | Yes | Yes | Yes | Yes | Yes | Yes |
| Influence of aestivation on the survival of Galba truncatula (Mollusca : Gasteropoda) populations according to altitude | Yes | Yes | Yes | Yes | Yes | Yes | Yes |
| Les réseaux de drainage superficiel et leur colonisation par Lymnaea truncatula Müller. A propos de quatre années d'observations en Haute-Vienne, France. In Annales de Recherches Vétérinaires | Yes | Yes | Yes | Yes | Yes | Yes | Yes |
| Life history traits variation in heterogeneous environment: The case of a freshwater snail resistance to pond drying | Yes | Yes | Yes | Yes | Yes | Yes | Yes |
| Local adaptation of the trematode Fasciola hepatica to the snail Galba truncatula | Yes | Yes | Yes | Yes | Yes | Yes | Yes |
| Metal bioaccumulation, oxidative stress, and biochemical alterations in the freshwater snail (Galba truncatula) exposed to municipal sewage | Yes | Yes | Yes | Yes | Yes | Yes | Yes |
| Rapid detection of Galba truncatula in water sources on pasture-land using loop-mediated isothermal amplification for control of trematode infections | Yes | Yes | Yes | Yes | Yes | Yes | Yes |
| The contamination of wild watercress with Fasciola hepatica in central France depends on the ability of several lymnaeid snails to migrate upstream towards the beds | Yes | Yes | Yes | Yes | Yes | Yes | Yes |
| The control of Galba truncatula (Gastropoda: Lymnaeidae) by the terrestrial snail Zonitoides nitidus on acid soils | Yes | Yes | Yes | Yes | Yes | Yes | Yes |
| The effects of low temperature on Lymnaea truncatula. | Yes | Yes | Yes | Yes | Yes | Yes | Yes |
| The performance of a PCR assay for field studies on the prevalence of Fasciola hepatica infection in Galba truncatula intermediate host snails | Yes | Yes | Can't tell | Yes | Yes | Yes | Yes |
| Validation of an interactive map assessing the potential spread of Galba truncatula as intermediate host of Fasciola hepaticain Switzerland | Yes | Yes | Yes | Yes | Yes | Yes | Yes |
| Vertical spatial behaviour patterns of Lymnaea truncatula in relation with origin of snails, infection with Fasciola hepatica, and experimental environment | Yes | Yes | Yes | Yes | Yes | Yes | Yes |
| **Quantitative Descriptive** | **Screening Questions** | | **Methodological Quality Criteria** | | | | |
| **Study Name** | **S1: Are there clear research questions?** | **S2: Do the collected data allow to address the research questions?** | **4.1** | **4.2** | **4.3** | **4.4** | **4.5** |
| A contribution to knowledge of freshwater molluscs (Mollusca) of the Krka River in the Krka National Park (Croatia) | Yes | Yes | Yes | Yes | Yes | Can't tell | Can't tell |
| A focus of Fasciola hepatica in Crete without human cases | Yes | Yes | Yes | Yes | Yes | Yes | Can't tell |
| A machine learning approach for modelling the occurrence of Galba truncatula as the major intermediate host for Fasciola hepatica in Switzerland | Yes | Yes | Yes | Yes | Yes | Can't tell | Yes |
| A new baseline for fascioliasis in Venezuela: lymnaeid vectors ascertained by DNA sequencing and analysis of their relationships with human and animal infection | Yes | Yes | Yes | Yes | Yes | Can't tell | Yes |
| A proposed ectochory of Galba truncatula snails between wallow sites enhances transmission of Fascioloides magna at gemenc, in Hungary | Yes | Yes | Yes | Yes | Yes | Can't tell | Can't tell |
| A Review of Species Diversity, Distribution and Ecology of Freshwater Gastropod Molluscs Inhabiting the Ukrainian Transcarpathian. | Yes | Yes | Yes | Yes | Yes | Can't tell | Can't tell |
| A snail on four continents: bird-mediated dispersal of a parasite vector. | Yes | Yes | Yes | Yes | Yes | Can't tell | Yes |
| An epidemiological study of Fasciola hepatica in the Netherlands. | Yes | Yes | Yes | Yes | Yes | Can't tell | Yes |
| An index to assess hydromorphological quality of Estonian surface waters based on macroinvertebrate taxonomic composition. | Yes | Yes | Yes | Yes | Yes | Can't tell | Yes |
| AN INTEGRATIVE APPROACH FOR THE IDENTIFICATION OF NATIVE AND EXOTIC LYMNAEIDS FROM BRAZIL | Yes | Yes | Yes | Yes | Yes | Can't tell | Can't tell |
| An interactive map to assess the potential spread of Lymnaea truncatula and the free-living stages of Fasciola hepatica in Switzerland | Yes | Yes | Yes | Yes | Yes | Can't tell | Yes |
| An investigation on the distribution of mollusc fauna of Lake Terkos (Istanbul/Turkey) related with some environmental parameters. | Yes | Yes | Yes | Yes | Yes | Can't tell | Yes |
| Aquatic molluscs in high mountain lakes of the Eastern Alps (Austria): Species-environment relationships and specific colonization behaviour | Yes | Yes | Yes | Yes | Yes | Can't tell | Yes |
| Biodiversity of aquatic gastropods in the Mont St-Michel basin (France) in relation to salinity and drying of habitats | Yes | Yes | Yes | Yes | Yes | Can't tell | Yes |
| Bioindication of ecotoxicity according to community structure of macrozoobenthic fauna | Yes | Yes | Yes | Yes | Yes | Can't tell | Can't tell |
| Bovine fasciolosis at increasing altitudes: Parasitological and malacological sampling on the slopes of Mount Elgon, Uganda | Yes | Yes | Yes | Yes | Yes | Can't tell | Yes |
| Changes in the Populations of Two Lymnaeidae and Their Infection by Fasciola hepatica and/or Calicophoron daubneyi over the Past 30 Years in Central France | Yes | Yes | Yes | Yes | Yes | Can't tell | Yes |
| Characterisation of fascioliasis lymnaeid intermediate hosts from Chile by DNA sequencing, with emphasis on Lymnaea viator and Galba truncatula | Yes | Yes | Yes | Yes | Yes | Can't tell | Yes |
| Contribution to the freshwater gastropods of the island of Andros in the Northern Cyclades (Aegean Islands, Greece) | Yes | Yes | Yes | Yes | Yes | Can't tell | Can't tell |
| Crenobiont, stygophile and stygobiont molluscs in the hydrographic area of the Trebišnjica River Basin | Yes | Yes | Yes | Yes | Yes | Can't tell | Can't tell |
| Cryptic intermediate snail host of the liver fluke Fasciola hepatica in Africa | Yes | Yes | Yes | Yes | Yes | Can't tell | Yes |
| Current decline in the number and size of Galba truncatula and Omphiscola glabra populations, intermediate hosts of Fasciola hepatica, on the acidic soils of Central France | Yes | Yes | Yes | Yes | Yes | Can't tell | Yes |
| Decline in the number and size of populations of two Lymnaeidae living in central France over the last decade | Yes | Yes | Yes | Yes | Yes | Can't tell | Yes |
| Determination of zones at risk for fasciolosis in the department of Haute-Vienne, central France: a retrospective study on naturalinfections detected in 108,481 Galba truncatula for 37 years | Yes | Yes | Yes | Yes | Yes | Yes | Yes |
| Discharge, substrate type and temperature as factors affecting gastropod assemblages in springs in northwestern Bosnia and Herzegovina | Yes | Yes | Yes | Yes | Yes | Can't tell | Yes |
| Distribution and habitats of the snail Lymnaea truncatula, intermediate host of the liver fluke Fasciola hepatica, in South Africa | Yes | Yes | Yes | Yes | Yes | Can't tell | Yes |
| Distribution and Protection of Mollusks in Latvia | Yes | Yes | Yes | Yes | Can't tell | Can't tell | Can't tell |
| Distribution of Lymnaeidae (Mollusca: Pulmonata), intermediate snail hosts of Fasciola hepatica in Venezuela | Yes | Yes | Yes | Yes | Yes | Can't tell | Yes |
| Distribution of Mollusca fauna in the streams of Tunceli Province (East Anatolia, Turkey) and its relationship with some physicochemical parameters | Yes | Yes | Yes | Yes | Yes | Can't tell | Yes |
| Diversity and ecology of molluscs (Gastropods) in mountain streams, Nurota mountain range, Uzbekistan | Yes | Yes | Yes | Yes | Yes | Can't tell | Yes |
| Diversity of Gastropoda in the Romanian sector of the Danube lower hydrographic basin | Yes | Yes | Yes | Yes | Yes | Can't tell | Can't tell |
| Ecological analysis of the composition and community structure of the Gorodnichanka river aquatic invertebrates (Grodno, Belarus) | Yes | Yes | Yes | Yes | Yes | Can't tell | Can't tell |
| Ecological basis of the distribution of the littoral freshwater molluscs in the vicinity of Tampere, South Finland | Yes | Yes | Yes | Yes | Yes | Can't tell | Yes |
| Ecological components and evolution of selfing in the freshwater snail Galba truncatula | Yes | Yes | Yes | Yes | Yes | Can't tell | Yes |
| Ecological Survey of Macroinvertebrate Communities in the Vrelska Padina and the Ivanštica Rivers (Eastern Serbia) | Yes | Yes | Yes | Yes | Yes | Can't tell | Yes |
| Ecology and species composition of Molluscs in upstream of the Kor River System, with two new records for the Fars Province, Iran | Yes | Yes | Yes | Yes | Yes | Can't tell | Yes |
| Effects of saline conditions and hydrologic permanence on snail assemblages in wetlands of Northeastern China. | Yes | Yes | Yes | Yes | Yes | Can't tell | Yes |
| Effects of Selection and Drift on G Matrix Evolution in a Heterogeneous Environment: A Multivariate Q(st)-F-st Test With the Freshwater Snail Galba truncatula | Yes | Yes | Yes | Yes | Yes | Can't tell | Yes |
| Endemic occurrence of Fasciola hepatica in an alpine ecosystem, Pyrenees, Northeastern Spain | Yes | Yes | Yes | Yes | Yes | Can't tell | Yes |
| Environmental and biotic factors affecting freshwater snail intermediate hosts in the Ethiopian Rift Valley region | Yes | Yes | Yes | Yes | Yes | Can't tell | Yes |
| Epidemiological studies on Fasciola hepatica in Gafsa oases (South West of Tunisia) | Yes | Yes | Yes | Yes | Yes | Yes | Yes |
| Fasciola hepatica and Paramphistomum daubneyi:changes in prevalences of natural infections in cattle and in Lymnaea truncatula from central France over the past 12 years | Yes | Yes | Yes | Yes | Yes | Yes | Yes |
| Fasciola hepatica infections in cattle and the freshwater snail Galba truncatula from Dakhla Oasis, Egypt | Yes | Yes | Yes | Yes | Yes | Yes | Yes |
| Fasciola hepatica infections in livestock flock, guanacos and coypus in two wildlife reserves in Argentina | Yes | Yes | Yes | Yes | Yes | Yes | Yes |
| Fasciola species and their vertebrate and snail intermediate hosts in East and Southern Africa: a review | Yes | Yes | Yes | Yes | Yes | Yes | Yes |
| Fauna of Mollusks of the Lower Reaches of the Danube River within the Territory of Ukraine | Yes | Yes | Yes | Yes | Can't tell | Can't tell | Can't tell |
| Fine-scale mapping of vector habitats using very high resolution satellite imagery: a liver fluke case-study | Yes | Yes | Yes | Yes | Yes | Can't tell | Yes |
| First investigations on the populations or Galba truncatula (Mollusca Gastropoda : Lyrunacidae), the snail host of Fasciola hepatica, in the irrigated district of Doukkala (northwestern Morocco). | Yes | Yes | Yes | Yes | Yes | Can't tell | Yes |
| First report of Fasciola larva infection in Galba truncatula (Müller, 1774)(Gastropoda, Lymnaeidae) occurring in the natural environment in Hokkaido, Japan. | Yes | Yes | Yes | Yes | Yes | Can't tell | Can't tell |
| First studies on the habitats of Galba truncatula (Mollusca Gastropoda : Lymnaeidae), the snail host of Fasciola hepatica, and the dynamics of snail populations in Northeastern Algeria | Yes | Yes | Yes | Yes | Yes | Can't tell | Yes |
| Freshwater gastropods of the western part of the Kola Peninsula and northern Karelia (northern Europe) | Yes | Yes | Yes | Yes | Yes | Can't tell | Can't tell |
| Freshwater mollusc biodiversity and conservation in two stressed Mediterranean basins | Yes | Yes | Yes | Yes | Yes | Can't tell | Yes |
| Freshwater molluscs in mountain lakes of the Eastern Alps (Austria): relationship between environmental variables and lake colonization | Yes | Yes | Yes | Yes | Yes | Can't tell | Yes |
| Freshwater molluscs of Kyrgyzstan with description of one new genus and species (Mollusca: Gastropoda) | Yes | Yes | Yes | Yes | Yes | Can't tell | Can't tell |
| Freshwater Snail Communities and Lake Classification. An Example from the Aland Islands, Southwestern Finland | Yes | Yes | Yes | Yes | Yes | Can't tell | Yes |
| Freshwater snails (Mollusca: Gastropoda) of Bulgaria: an updated annotated checklist. | Yes | Yes | Yes | Yes | Yes | Can't tell | Can't tell |
| Freshwater snails as the intermediate host of trematodes in Iran: a systematic review | Yes | Yes | Yes | Yes | Yes | Yes | Yes |
| Further Observations on the Life-history of Limnaea truncatula | Yes | Yes | Yes | Yes | Yes | Can't tell | Can't tell |
| Galba truncatula (Gastropoda, Lymnaeidae): effects of daily waterlevel variations on the ecology and ethology of populations living upstream from a dam | Yes | Yes | Yes | Yes | Yes | Yes | Yes |
| Galba truncatula (Gastropoda, Lymnaeidae): First findings on populations showing a single annual generation in lowland zones of central France | Yes | Yes | Yes | Yes | Yes | Yes | Yes |
| Galba truncatula (Muller, 1774). A factor for spreading of fasciolosis. | Yes | Yes | Yes | Yes | Yes | Can't tell | Can't tell |
| Galba truncatula and Fasciola hepatica: Genetic costructures and interactions with intermediate host dispersal | Yes | Yes | Yes | Yes | Yes | Yes | Yes |
| Galba truncatula and Omphiscola glabra (Gastropoda, Lymnaeidae): present decline in populations living on sedimentary soils in central France | Yes | Yes | Yes | Yes | Yes | Can't tell | Yes |
| Gastropod Species Distribution and its Relation with some Physico-chemical Parameters of the Malatya's Streams (East Anatolia, Turkey) | Yes | Yes | Yes | Yes | Yes | Can't tell | Yes |
| Genetic uniformity, geographical spread and anthropogenic habitat modifications of lymnaeid vectors found in a One Health initiative in the highest human fascioliasis hyperendemic of the Bolivian Altiplano | Yes | Yes | Yes | Yes | Yes | Can't tell | Yes |
| Growth, Laying, and Survival Rates of the Galba truncatula Snails Infected with Fasciola hepatica | Yes | Yes | Yes | Yes | Yes | Can't tell | Yes |
| Habitat suitability modelling for predicting potential habitats of freshwater snail intermediate hosts in Omo-Gibe river basin, Southwest Ethiopia | Yes | Yes | Yes | Yes | Yes | Can't tell | Yes |
| Habitats of Limnaea truncatula in England and Wales during Dry Seasons | Yes | Yes | Yes | Yes | Yes | Can't tell | Can't tell |
| HABITATS OF LYMNAEA-TRUNCATULA, INTERMEDIATE HOST OF FASCIOLA-HEPATICA | Yes | Yes | Yes | Yes | Yes | Can't tell | Yes |
| High quantitative and no molecular differentiation of a freshwater snail (Galba truncatula) between temporary and permanent water habitats | Yes | Yes | Yes | Yes | Yes | Yes | Yes |
| Hydrobiological particularities of Maglavit Lake (Romania)–the place and role of Gastropod populations | No | Can't tell | Can't tell | Yes | Yes | Can't tell | Can't tell |
| Hydrographic network structure and population genetic differentiation in a vector of fasciolosis, Galba truncatula | Yes | Yes | Yes | Yes | Yes | Yes | Yes |
| Identification of factors associated with Fasciola hepatica infection risk areas on pastures via an environmental DNA survey of Galba truncatula distribution using droplet digital and quantitative real-time PCR assays | Yes | Yes | Yes | Yes | Yes | Can't tell | Yes |
| Identification of viable fluke metacercarial challenge to livestock | Yes | Yes | Yes | Yes | Yes | Can't tell | Yes |
| Interaction between the intermediate host of Fascioliasis in Tunisia, Galba truncatula and a possible competitor, Melanoides tuberculata (Muller, 1774): a field study | Yes | Yes | Yes | Yes | Yes | Can't tell | Yes |
| Lack of molluscan host diversity and the transmission of an emerging parasitic disease in Bolivia | Yes | Yes | Yes | Yes | Yes | Yes | Yes |
| Larval trematode infections in Galba truncatula (Gastropoda, Lymnaeidae) from the Brenne Regional Natural Park, central France | Yes | Yes | Yes | Yes | Yes | Can't tell | Yes |
| Life history and population fluctuations of Lymnaea truncatula (Mull), the snail vector of fascioliasis. | Yes | Yes | Yes | Yes | Yes | Can't tell | Yes |
| Liver Rot of Sheep, and Bionomics of Limnaea truncatula in the Aberystwyth Area | Can't tell | Can't tell | Can't tell | Yes | Yes | Can't tell | Can't tell |
| Longitudinal study on the temporal and micro-spatial distribution of Galba truncatula in four farms in Belgium as a base for small-scale risk mapping of Fasciola hepatica | Yes | Yes | Yes | Yes | Yes | Yes | Yes |
| Lymnaea schirazensis, an Overlooked Snail Distorting Fascioliasis Data: Genotype, Phenotype, Ecology, Worldwide Spread, Susceptibility, Applicability | Yes | Yes | Yes | Yes | Yes | Yes | Yes |
| Lymnaea truncatula MULLER, 1774 (Pulmonata: Lymnaeidae) infected with Fasciola hepática (Linnaeus, 1758)(Trematoda: Digenea), in Moscow districts, Russian Federation. | Yes | Yes | Yes | Yes | Yes | Can't tell | Yes |
| Lymnaea truncatula, intermediate host of some Plagiorchiidae and Notocotylidae species in Leon, NW Spain | Yes | Yes | Yes | Yes | Yes | Can't tell | Yes |
| Lymnaeid habitats in swampy meadows on acid soil: Effects of agricultural impacts. | Yes | Yes | Yes | Yes | Yes | Can't tell | Yes |
| Lymnaeidae from Santander and bordering departments of Colombia: Morphological characterization, molecular identification and natural infection with Fasciola hepatica | Yes | Yes | Yes | Yes | Yes | Can't tell | Yes |
| Lymnea truncatula (Mollusca Lymnaeidae) in the rice fields of eastern Spain. | Yes | Yes | Yes | Yes | Yes | Can't tell | Yes |
| Malacofauna of Svityaz and Pisochne Lakes in Shatsk National Nature Park and the role of molluscs in formation of cercarial dermatitis nidi | Yes | Yes | Yes | Yes | Yes | Can't tell | Yes |
| Malacological survey in a bottle of water: A comparative study between manual sampling and environmental DNA metabarcoding approaches. | Yes | Yes | Yes | Yes | Yes | Can't tell | Yes |
| Microsatellites in the hermaphroditic snail, Lymnaea truncatula, intermediate host of the liver fluke, Fasciola hepatica | Yes | Yes | Yes | Yes | Yes | Yes | Yes |
| Molecular characterisation of Galba truncatula, Lymnaea neotropica and L. schirazensis from Cajamarca, Peru and their potential role in transmission of human and animal fascioliasis | Yes | Yes | Yes | Yes | Yes | Yes | Yes |
| Molecular characterization of cryptic and sympatric lymnaeid species from the Galba/Fossaria group in Mendoza Province, Northern Patagonia, Argentina | Yes | Yes | Yes | Yes | Yes | Can't tell | Yes |
| Molecular detection of the infection with Fasciola hepatica in field-collected snails of Galba truncatula and Lymnaea stagnalis from West Azarbaijan, Iran. | Yes | Yes | Yes | Yes | Yes | Can't tell | Yes |
| Molecular Evolution of Freshwater Snails with Contrasting Mating Systems | Yes | Yes | Yes | Yes | Yes | Can't tell | Yes |
| Mollusc communities along upstream–downstream gradients in small coastal basins of the south-western Iberian Peninsula | Yes | Yes | Yes | Yes | Yes | Can't tell | Yes |
| Molluscs of selected watercourses and reservoirs in Vilnius | Yes | Yes | Yes | Yes | Yes | Can't tell | Can't tell |
| Mollusks (Gastropoda) as intermediate hosts of cattles’ trematodes (Trematoda) in conditions of Dnipro basin’s small ponds (Northern Ukraine) | Yes | Yes | Yes | Yes | Yes | Can't tell | Can't tell |
| Mollusks biodiversity of Lake Sevan, Armenia | Yes | Yes | Yes | Yes | Yes | Can't tell | Can't tell |
| Natural infection of Lymnaea truncatula by the liver fluke Fasciola hepatica in the Porma Basin, León, NW Spain | Yes | Yes | Yes | Yes | Yes | Can't tell | Yes |
| New land snails from Montenegro and Albania | Can't tell | Can't tell | Can't tell | Yes | Yes | Can't tell | Can't tell |
| Non-marine aquatic molluscs (Gastropoda, Bivalvia) of Rab Island (Croatia) | Yes | Yes | Yes | Yes | Yes | Can't tell | Can't tell |
| Notes on the continental malacofauna of Rhodes, with two new species for the fauna of the island. | Yes | Yes | Yes | Yes | Yes | Can't tell | Can't tell |
| Notes on the ecology and species diversity of the inland molluscs of Samothraki Island (North-Eastern Greece) | Yes | Yes | Yes | Yes | Yes | Can't tell | Can't tell |
| Occurrence of Fasciola (Digenea: Fasciolidae) Species in Livestock, Wildlife and Humans, and the Geographical Distribution of Their Intermediate Hosts in South Africa-A Scoping Review | Yes | Yes | Yes | Yes | Yes | Yes | Yes |
| One Health initiative in the Bolivian Altiplano human fascioliasis hyperendemic area: Lymnaeid biology, population dynamics, microecology and climatic factor influences | Yes | Yes | Yes | Yes | Yes | Can't tell | Yes |
| Patterns of Fasciola hepatica infection in Danish dairy cattle: implications for on-farm control of the parasite based on different diagnostic methods | Yes | Yes | Yes | Yes | Yes | Yes | Yes |
| Phylogeography and genetic divergence of some lymnaeid snails, intermediate hosts of human and animal fascioliasis with special reference to lymnaeids from the Bolivian Altiplano | Yes | Yes | Yes | Yes | Yes | Yes | Yes |
| Physico-chemical parameters and benthic macroinvertebrates of Ogunpa river at Bodija, Ibadan, Oyo State. | Yes | Yes | Yes | Yes | Yes | Can't tell | Can't tell |
| Potential Hybridization of Fasciola hepatica and F. gigantica in Africa-A Scoping Review | Yes | Yes | Yes | Yes | Yes | Yes | Yes |
| Prevalence of Fasciola hepatica in Galba truncatula detected by Multiplex PCR in the province of El Tarf (Algeria) | Yes | Yes | Yes | Yes | Yes | Can't tell | Yes |
| Prevalence of Fasciola hepatica in the intermediate host Lymnaea truncatula detected by real time TaqMan PCR in populations from 70 Swiss farms with cattle husbandry | Yes | Yes | Yes | Yes | Yes | Can't tell | Yes |
| Prevalence of Fascioloides magna in Galba truncatula in the Danube backwater area east of Vienna, Austria. | Yes | Yes | Yes | Yes | Yes | Can't tell | Can't tell |
| Radular variations in freshwater snails of the family Lymnaeidae (Mollusca: Gastropoda: Basommatophora) from northwestern Iran | Yes | Yes | Yes | Yes | Yes | Can't tell | Can't tell |
| Real-time PCR strategy for rapid discrimination among main lymnaeid species from Argentina | Yes | Yes | Yes | Yes | Yes | Can't tell | Can't tell |
| Recovery of Fascioloides magna (Digenea) population in spite of treatment programme? Screening of Galba truncatula (Gastropoda, Lymnaeidae) from Lower Austria | Yes | Yes | Yes | Yes | Yes | Can't tell | Yes |
| Relationships between the amphibious behaviour of the snail Galba truncatula (Mollusca Gastropoda : Lymnaeidae), altitude, and the type of its habitats in central Morocco | Yes | Yes | Yes | Yes | Yes | Can't tell | Yes |
| Relationships between the distribution of Galba truncatula (Gastropoda: Lymnaeidae) climatic conditions and the altitude of municipalities in Haute Vienne (France) | Yes | Yes | Yes | Yes | Yes | Yes | Yes |
| Ribosomal DNA ITS-1 sequencing of Galba truncatula (Gastropoda, Lymnaeidae) and its potential impact on fascioliasis transmission in Mendoza, Argentina | Yes | Yes | Yes | Yes | Yes | Yes | Can't tell |
| Schistosomatidae from the trematode fauna of aquatic and semi-aquatic birds in Uzbekistan | Yes | Yes | Yes | Yes | Yes | Can't tell | Can't tell |
| Scientific Results of the Yale North India Expedition. Biological Report No. 21 Aquatic and Amphibious Molluscs | Can't tell | Can't tell | Can't tell | Yes | Can't tell | Can't tell | Can't tell |
| Seasonal variations in the microclimate of Lymnaea truncatula habitats | Yes | Yes | Yes | Yes | Yes | Yes | Yes |
| Seasonal variations of Fasciola hepatica infection in goats in the area of Haouz (Morocco). | Yes | Yes | Yes | Yes | Yes | Yes | Can't tell |
| Semi-naturally managed forests support diverse land snail assemblages in Estonia | Yes | Yes | Yes | Yes | Yes | Can't tell | Yes |
| Small effective population sizes in a widespread selfing species, Lymnaea truncatula (Gastropoda: Pulmonata) | Yes | Yes | Yes | Yes | Yes | Yes | Yes |
| Soil type and the distribution of Lymnaea truncatula. | Yes | Yes | Yes | Yes | Yes | Can't tell | Can't tell |
| Spatio-temporal distribution of freshwater snail species in relation to migration and environmental factors in an irrigated area from Morocco | Yes | Yes | Yes | Yes | Yes | Can't tell | Yes |
| Species composition of freshwater snail communities in lakes of southern and western Finland | Yes | Yes | Can't tell | Yes | Yes | Can't tell | Yes |
| Species-area relationships, water chemistry and species turnover of freshwater snails on the Åland Islands, southwestern Finland. | Yes | Yes | Yes | Yes | Yes | Can't tell | Yes |
| Sporadic notes on the land snail (Gastropoda) fauna of the North Bihor Mountains, Romania | Yes | Yes | Yes | Yes | Yes | Can't tell | Can't tell |
| Spread of the fascioliasis endemic area assessed by seasonal follow-up of rDNA ITS-2 sequenced lymnaeid populations in Cajamarca, Peru | Yes | Yes | Yes | Yes | Yes | Can't tell | Yes |
| Standard faunistical work on the molluscs of Codru-Moma Mountains (Romania). | Yes | Yes | Yes | Yes | Yes | Can't tell | Can't tell |
| Study on the Vector Role for Calicophoron daubneyi of Some Aquatic Snails form Western Romania | Yes | Yes | Yes | Yes | Yes | Can't tell | Can't tell |
| Survey and population dynamics of freshwater snails in newly settled areas of the Sinai Peninsula. | Yes | Yes | Yes | Yes | Yes | Can't tell | Yes |
| Synopsis of the Egyptian freshwater snail fauna | Yes | Yes | Yes | Yes | Yes | Yes | Can't tell |
| Systematic descriptions and seasonal variations of mollusc in Chandigarh (U.T., India) and its surrounding freshwater bodies | Yes | Yes | Yes | Yes | Yes | Can't tell | Can't tell |
| Temporal dynamics of trematode intermediate snail host environmental DNA in small water body habitats | Yes | Yes | Yes | Yes | Yes | Yes | Yes |
| Temporal studies on Fasciola hepatica in Galba truncatula in the west of Ireland | Yes | Yes | Yes | Yes | Yes | Can't tell | Yes |
| The characteristics of habitats colonized by three species of Lymnaea (Mollusca) in swampy meadows on acid soil: their interest for control of fasciolosis | Yes | Yes | Yes | Yes | Yes | Can't tell | Yes |
| The detection of snail host habitats in liver fluke infected farms by use of plant indicators | Yes | Yes | Yes | Yes | Yes | Can't tell | Yes |
| The distribution of Fasciola hepatica and Fasciola gigantica within southern Tanzania - constraints associated with the intermediate host | Yes | Yes | Yes | Yes | Yes | Can't tell | Yes |
| The ecological complex role of the macroinvertebrate fauna from the River Ciric (Iași, România). | Yes | Yes | Yes | Yes | Yes | Can't tell | Can't tell |
| The finding of North American freshwater gastropods of the genus Planorbella Haldeman, 1842 (Pulmonata: Planorbidae) in East Siberia | Yes | Yes | Yes | Yes | Yes | Can't tell | Can't tell |
| The freshwater molluscs from the Târnava rivers basin (Transylvania, Romania). | Yes | Yes | Yes | Yes | Yes | Can't tell | Can't tell |
| The habitats of Lymnaea truncatula Muller (Mollusca) along two river banks | Yes | Yes | Yes | Yes | Yes | Can't tell | Yes |
| The Life-History of Limnaea truncatula under Laboratory Conditions. | Yes | Yes | Yes | Yes | Yes | Can't tell | Can't tell |
| The Mollusk Fauna of Istranca Stream (Terkos-Istanbul) And Some Physico-Chemical Parameters of Their Abundance | Yes | Yes | Yes | Yes | Yes | Can't tell | Yes |
| The mollusk fauna of Lake Sapanca (Turkey: Marmara) and some physico-chemical parameters of their abundance. | Yes | Yes | Yes | Yes | Yes | Can't tell | Yes |
| The natural watercress beds in the region of Limousin (France). About of some observations on numerical variations of Lymnaeid snails | Yes | Yes | Yes | Yes | Yes | Yes | Yes |
| The New Red List of the molluscs of Latvia | Yes | Yes | Yes | Yes | Can't tell | Yes | Can't tell |
| The Northern Bolivian Altiplano: a region highly endemic for human fascioliasis | Yes | Yes | Yes | Yes | Yes | Can't tell | Can't tell |
| The prevalence and development of digenean parasites within their intermediate snail host, Galba truncatula, in a geographic area where the presence of Calicophoron daubneyi has recently been confirmed | Yes | Yes | Yes | Yes | Yes | Yes | Yes |
| The relationship between the size of Lymnaea truncatula naturally infected with Fasciola hepatica and the intensity and maturity of the redial infection. | Yes | Yes | Yes | Yes | Yes | Yes | Yes |
| THE STUDIES OF MOLLUSCS IN MORICSALA STRICT NATURE RESERVE AND OTHER SITES IN WESTERN LATVIA. | Yes | Yes | Yes | Yes | Yes | Can't tell | Can't tell |
| The Worm Burden of Sheep on Improved and Unimproved Hill Pastures. | Yes | Yes | Yes | Yes | Yes | Can't tell | Can't tell |
| Towards assessing fine-scale indicators for the spatial transmission risk of Fasciola hepatica in cattle | Yes | Yes | Yes | Yes | Yes | Can't tell | Yes |
| Transmission of Calicophoron daubneyi and Fasciola hepatica in Galicia (Spain): Temporal follow-up in the intermediate and definitive hosts | Yes | Yes | Yes | Yes | Yes | Yes | Yes |
| Transmission patterns of Fasciola hepatica to ruminants in Sweden | Yes | Yes | Yes | Yes | Yes | Yes | Yes |
| **Mixed Methods (all 3+4)** | **Screening Questions** | | **Methodological Quality Criteria** | | | | |
| **Study Name** | **S1: Are there clear research questions?** | **S2: Do the collected data allow to address the research questions?** | **3.1** | **3.2** | **3.3** | **3.4** | **3.5** |
| Donkey Fascioliasis Within a One Health Control Action: Transmission Capacity, Field Epidemiology, and Reservoir Role in a Human Hyperendemic Area | Yes | Yes | Yes | Yes | Yes | Yes | Yes |
|  |  |  | **4.1** | **4.2** | **4.3** | **4.4** | **4.5** |
|  |  |  | Yes | Yes | Yes | Yes | Yes |
|  |  |  | **5.1** | **5.2** | **5.3** | **5.4** | **5.5** |
|  |  |  | Yes | Yes | Yes | Yes | Yes |

| Equines as reservoirs of human fascioliasis: transmission capacity, epidemiology and pathogenicity in Fasciola hepatica-infected mules | Yes | Yes | **3.1** | **3.2** | **3.3** | **3.4** | **3.5** |
| --- | --- | --- | --- | --- | --- | --- | --- |
|  |  |  | Yes | Yes | Yes | Yes | Yes |
|  |  |  | **4.1** | **4.2** | **4.3** | **4.4** | **4.5** |
|  |  |  | Yes | Yes | Yes | Yes | Yes |
|  |  |  | **5.1** | **5.2** | **5.3** | **5.4** | **5.5** |
|  |  |  | Yes | Yes | Yes | Yes | Yes |
| Fascioliasis in Llama, Lama glama, in Andean Endemic Areas: Experimental Transmission Capacity by the High Altitude Snail Vector Galba truncatula and Epidemiological Analysis of Its Reservoir Role | Yes | Yes | **3.1** | **3.2** | **3.3** | **3.4** | **3.5** |
|  |  |  | Yes | Yes | Yes | Yes | Yes |
|  |  |  | **4.1** | **4.2** | **4.3** | **4.4** | **4.5** |
|  |  |  | Yes | Yes | Yes | Yes | Yes |
|  |  |  | **5.1** | **5.2** | **5.3** | **5.4** | **5.5** |
|  |  |  | Yes | Yes | Yes | Yes | Yes |
| Studies on the Life-Cycle of Fasciola Hepatica (Linnaeus) and of Its Snail Host, Limnaea (Galba) Truncatula (Müller), in the Field and Under Controlled Conditions in the Laboratory | Yes | Yes | **3.1** | **3.2** | **3.3** | **3.4** | **3.5** |
|  |  |  | Yes | Yes | Yes | Yes | Yes |
|  |  |  | **4.1** | **4.2** | **4.3** | **4.4** | **4.5** |
|  |  |  | Yes | Yes | Yes | Yes | Yes |
|  |  |  | **5.1** | **5.2** | **5.3** | **5.4** | **5.5** |
|  |  |  | Yes | Yes | Yes | Yes | Yes |
| Variability of Fasciola infections in Lymnaea truncatula as a function of snail generation and snail activity. Journal of helminthology | Yes | Yes | **3.1** | **3.2** | **3.3** | **3.4** | **3.5** |
|  |  |  | Yes | Yes | Yes | Yes | Yes |
|  |  |  | **4.1** | **4.2** | **4.3** | **4.4** | **4.5** |
|  |  |  | Yes | Yes | Yes | Yes | Yes |
|  |  |  | **5.1** | **5.2** | **5.3** | **5.4** | **5.5** |
|  |  |  | Yes | Yes | Yes | Yes | Yes |

Table S4: Summary statistics of each study used.
NOTE 1: This study was, in part, a literature review which found literature mentioning *G. truncatula* populations in several countries. These populations were noted on a map. However, as the position of some of the populations and the borders were unclear, only countries explicitly mentioned in tables, figures and the text were included in this study.

| **Study Title** | **Authors** | **Year of Publication** | **Nation(s) of origin of snail data** | **Study Type** | **Used in Per Study Count** | **Used in Individual Habitat Count** |
| --- | --- | --- | --- | --- | --- | --- |
| A contribution to knowledge of freshwater molluscs (Mollusca) of the Krka River in the Krka National Park (Croatia) | Beran, L | 2016 | Croatia | Small-Scale Survey | Yes | Yes |
| A focus of Fasciola hepatica in Crete without human cases | Antoniou, M; Lionis, C; and Tselentis, Y | 1997 | Greece | Small-Scale Survey | Yes | No |
| A machine learning approach for modelling the occurrence of Galba truncatula as the major intermediate host for Fasciola hepatica in Switzerland | Roessler, AS; Oehm, AW; Knubben-Schweizer, G and Groll, A | 2022 | Switzerland | Large-Scale Survey | Yes | No |
| A new baseline for fascioliasis in Venezuela: lymnaeid vectors ascertained by DNA sequencing and analysis of their relationships with human and animal infection | Bargues, MD; González, L; Artigas, P and Mas-Coma, S | 2011 | Venezuela | Small-Scale Survey | Yes | Yes |
| A new method for laboratory rearing of Galba truncatula, the intermediate host of Fasciola hepatica | Moazeni, M; Ahmadi, A and Alavi, AM | 2018 | Iran | Lab-Based Study | Yes | Yes |
| A proposed ectochory of Galba truncatula snails between wallow sites enhances transmission of Fascioloides magna at gemenc, in Hungary | Juhász, A and Majoros, G | 2023 | Hungary | Small-Scale Survey | Yes | Yes |
| A retrospective study on the metacercarial production of Fasciola hepatica from experimentally infected Galba truncatula in central France. | Dreyfuss, G; Alarion, N; Vignoles, P and Rondelaud, D | 2006 | France | Lab-Based Study | Yes | Yes |
| A Review of Species Diversity, Distribution and Ecology of Freshwater Gastropod Molluscs Inhabiting the Ukrainian Transcarpathian. | Anistratenko, VV; Furyk, YI; Anistratenko, OY and Degtyarenko, EV | 2019 | Ukraine | Small-Scale Survey | Yes | Yes |
| A snail on four continents: bird-mediated dispersal of a parasite vector. | Van Leeuwen, CH; van der Velde, G., Wouter, H; Wisselink, TVA; Sablon, R and Johannes, HP; | 2012 | Netherlands, Iceland, Norway, United Kingdom, Russia, France, Italy, Spain, Portugal, Greece, Austria, India, Morocco, Yemen, Venezuela, Kenya, Democratic Republic of Congo, Peru, Bolivia, Argentina, Chile, United States and Tunisia SEE NOTE 1 | Lab-Based Study | No | No |
| A strategic dosing scheme for the control of fasciolosis in cattle and sheep in Ireland | Parr, SL and Gray, JS | 2000 | Republic of Ireland | Field Experiment | Yes | No |
| A survey of the Lymnaea truncatula habitat areas in Co Down, Northern Ireland | Ross, JG and Taylor, SM | 1970 | United Kingdom | Small-Scale Survey | No | No |
| A survey on the Lymnaea truncatula habitat areas in Co. Tyrone, and Co. Fermanagh, Northern Ireland. | Taylor, SM and Ross, JG | 1970 | United Kingdom | Small-Scale Survey | No | No |
| Allopatric combination of Fasciola hepatica and Lymnaea truncatula is more efficient than sympatric ones. | Gasnier, N; Rondelaud, D., Abrous, M; Carreras, F; Boulard, C; Diez-Banos, P and Cabaret, J | 2000 | France and Spain | Lab-Based Study | No | No |
| An epidemiological study of Fasciola hepatica in the Netherlands. | Gaasenbeek, CPH; Over, HJ; Noorman, N and De Leeuw, WA | 1992 | Netherlands | Small-Scale Survey | Yes | No |
| An index to assess hydromorphological quality of Estonian surface waters based on macroinvertebrate taxonomic composition. | Timm, H, Käiro, K; Möls, T and Virro, T | 2011 | Estonia | Large-Scale Survey | No | No |
| An integrative approach for the identification of native and exotic lymnaeids from Brazil | Medeiros, C., Scholte, LLS; Cardoso, PCM; Pointier, JP; Rumi, A., Oliveira, IHR; de Souza, PM D'ávila, S; Rosenberg, G; dos Santos Carvalho, O and Caldeira, RL | 2022 | Brazil | Small-Scale Survey | No | No |
| An interactive map to assess the potential spread of Lymnaea truncatula and the free-living stages of Fasciola hepatica in Switzerland | Rapsch, C; Dahinden, T; Heinzmann, D; Torgerson, PR; Braun, U; Deplazes, P; Hurni, L; Bär, H and Knubben-Schweizer, G | 2008 | Switzerland | Literature Review | No | No |
| An investigation on the distribution of mollusc fauna of Lake Terkos (Istanbul/Turkey) related with some environmental parameters. | Sahin, SK | 2012 | Turkey | Small-Scale Survey | Yes | No |
| Aplexa hypnorum (Gastropoda: Physidae) exerts competition on two lymnaeid species in periodically dried ditches | Rondelaud, D; Vignoles, P and Dreyfuss, G | 2016 | France | Field and Lab Study | Yes | Yes |
| Aquatic molluscs in high mountain lakes of the Eastern Alps (Austria): Species-environment relationships and specific colonization behaviour | Sturm, R | 2012 | Austria | Small-Scale Survey | Yes | Yes |
| Biodiversity of aquatic gastropods in the Mont St-Michel basin (France) in relation to salinity and drying of habitats | Costil, K; Dussart, GBJ and Daguzan, J | 2001 | France | Small-Scale Survey | Yes | No |
| Bioindication of ecotoxicity according to community structure of macrozoobenthic fauna | Bubinas, A and Jagminienė, I | 2001 | Lithuania | Small-Scale Survey | Yes | Yes |
| Bionomics of Limnaea truncatula and the Parthenitae of Fasciola hepatica under Drought Conditions | Kendall, SB | 1949 | United Kingdom | Lab-Based Study | No | No |
| Bovine fasciolosis at increasing altitudes: Parasitological and malacological sampling on the slopes of Mount Elgon, Uganda | Howell, A; Mugisha, L; Davies, J; LaCourse, EJ; Claridge, J; Williams, DJL; Kelly-Hope, L; Betson, M; Kabatereine, NB; Stothard, JR | 2012 | Uganda | Small-Scale Survey | Yes | No |
| Changes in the Populations of Two Lymnaeidae and Their Infection by Fasciola hepatica and/or Calicophoron daubneyi over the Past 30 Years in Central France | Rondelaud, D; Vignoles, P and Dreyfuss, G | 2022 | France | Large-Scale Survey | Yes | No |
| Characterisation of fascioliasis lymnaeid intermediate hosts from Chile by DNA sequencing, with emphasis on Lymnaea viator and Galba truncatula | Artigas, P; Bargues, MD; y Sierra, RLM; Agramunt, VH and Mas-Coma, S | 2011 | Chile | Field and Lab Study | No | No |
| Characteristics of Fasciola hepatica infections in Galba truncatula originating from riverbank populations | Vignoles, P; Rondelaud, D and Dreyfuss, G | 2011 | France | Lab-Based Study | Yes | Yes |
| Comparative strategies and success of sympatric and allopatric Fasciola hepatica infecting Galba truncatula of different susceptibilities | Sanabria, R; Mouzet, R; Courtioux, B; Vignoles, P; Rondelaud, D; Dreyfuss, G; Cabaret, J and Romero, J | 2013 | France | Lab-Based Study | Yes | Yes |
| Consequences of invasion by Pseudosuccinea columella on the dynamics of native lymnaeids living on the acid soils of central France | Vignoles, P; Dreyfuss, G and Rondelaud, D | 2018 | France | Field and Lab Study | Yes | Yes |
| Contribution to the freshwater gastropods of the island of Andros in the Northern Cyclades (Aegean Islands, Greece) | Georgopoulou, E., Gloer, P. and Simaiakis, SM | 2016 | Greece | Small-Scale Survey | Yes | Yes |
| Correlation between parasite prevalence and adult size in a trematode-mollusc system: evidence for evolutionary gigantism in the freshwater snail Galba truncatula? | Chapuis, E | 2009 | Switzerland | Lab-Based Study | No | No |
| Crenobiont, stygophile and stygobiont molluscs in the hydrographic area of the Trebišnjica River Basin | Falniowski, A; Lewarne, B; Rysiewska, A; Osikowski, A and Hofman, S | 2021 | Bosnia and Herzegovina and Croatia | Small-Scale Survey | Yes | Yes |
| Cryptic intermediate snail host of the liver fluke Fasciola hepatica in Africa | Mahulu, A; Clewing, C; Stelbrink, B; Chibwana, FD; Tumwebaze I; Russell Stothard, J and Albrecht C. | 2019 | Morocco, Germany, Greece, Russia, Slovenia, Nepal and France | Lab-Based Study | No | No |
| Current decline in the number and size of Galba truncatula and Omphiscola glabra populations, intermediate hosts of Fasciola hepatica, on the acidic soils of Central France | Dreyfuss, G; Vignoles, P and Rondelaud, D | 2016 | France | Large-Scale Survey | Yes | Yes |
| Decline in the number and size of populations of two Lymnaeidae living in central France over the last decade | Vignoles, P; Dreyfuss, G and Rondelaud, D | 2022 | France | Large-Scale Survey | Yes | No |
| Detection of Galba truncatula, Fasciola hepatica and Calicophoron daubneyi environmental DNA within water sources on pasture land, a future tool for fluke control? | Jones, RA; Brophy, PM; Davis, CN; Davies, TE; Emberson, H; Stevens, PR and Williams, HW | 2018 | United Kingdom | Field and Lab Study | Yes | No |
| Determination of zones at risk for fasciolosis in the department of Haute-Vienne, central France: a retrospective study on naturalinfections detected in 108,481 Galba truncatula for 37 years | Vignoles, P., Rondelaud, D. and Dreyfuss, G. | 2017 | France | Literature Review | Yes | Yes |
| Diel activity cycles of freshwater gastropods under natural light: Patterns and ecological implications | Lombardo, P; Miccoli, FP; Giustini, M and Cicolani, B | 2010 | Italy | Lab-Based Study | No | No |
| Discharge, substrate type and temperature as factors affecting gastropod assemblages in springs in northwestern Bosnia and Herzegovina | Dmitrović, D; Savić, A. and Pešić, V | 2016 | Bosnia and Herzegovina | Small-Scale Survey | Yes | Yes |
| Distribution and habitats of the snail Lymnaea truncatula, intermediate host of the liver fluke Fasciola hepatica, in South Africa | De Kock, KN; Wolmarans, CT and Bornman, M | 2003 | South Africa and Lesotho | Large-Scale Survey | Yes | Yes |
| Distribution and Protection of Mollusks in Latvia | Rudzite, M; Boikova, E; Dreijers, E; Jakubane, I; Jermakovs, V; Parele, E; Pilate, D and Rudzitis, M | 2018 | Latvia | Literature Review | No | No |
| Distribution of Lymnaeidae (Mollusca: Pulmonata), intermediate snail hosts of Fasciola hepatica in Venezuela | Pointier, JP; Noya, O; Alarcon de Noya, B and Theron, A | 2009 | Venezuela | Large-Scale Survey | Yes | Yes |
| Distribution of Mollusca fauna in the streams of Tunceli Province (East Anatolia, Turkey) and its relationship with some physicochemical parameters | Şahin, SK and Zeybek, M | 2020 | Turkey | Small-Scale Survey | Yes | Yes |
| Diversity and ecology of molluscs (Gastropods) in mountain streams, Nurota mountain range, Uzbekistan | Kudratov, J; Pazilov, A; Maxammadoyev, Z; Urazova, R; Otakulov, B; Barazov, B; Keldiyarov, K; Soatova, Z and Urinova, X. | 2023 | Uzbekistan | Small-Scale Survey | Yes | Yes |
| Diversity of Gastropoda in the Romanian sector of the Danube lower hydrographic basin | Cioboiu, O | 2006 | Romania | Literature Review | No | No |
| Donkey Fascioliasis Within a One Health Control Action: Transmission Capacity, Field Epidemiology, and Reservoir Role in a Human Hyperendemic Area | Mas-Coma, S; Buchon, P; Funatsu, IR; Angles, R; Mas-Bargues, C; Artigas, P; Valero, MA and Bargues, MD | 2020 | Bolivia | Field and Lab Study | No | No |
| Ecological analysis of the composition and community structure of the Gorodnichanka river aquatic invertebrates (Grodno, Belarus) | Phyzhaya, AV and Yanchurevich, OV | 2015 | Belarus | Small-Scale Survey | Yes | Yes |
| Ecological basis of the distribution of the littoral freshwater molluscs in the vicinity of Tampere, South Finland | Aho, J | 1966 | Finland | Large-Scale Survey | Yes | Yes |
| Ecological components and evolution of selfing in the freshwater snail Galba truncatula | Trouve, S; Degen, L and Goudet, J | 2005 | Switzerland | Field Experiment | Yes | No |
| Ecological Survey of Macroinvertebrate Communities in the Vrelska Padina and the Ivanštica Rivers (Eastern Serbia) | Đuknić, J; Bjelanović, K; Durutović, A and Jovanović, V | 2010 | Serbia | Small-Scale Survey | Yes | Yes |
| Ecology and species composition of Molluscs in upstream of the Kor River System, with two new records for the Fars Province, Iran | Abbaspour, F; Yaripour, S; Gloeer, P and Zamanpoore, M | 2019 | Iran | Small-Scale Survey | Yes | Yes |
| Effects of Pirimicarb carbamate insecticide alone and in combination with lead (Pb) on biochemical parameters of soft tissues in freshwater snail, Galba truncatula | Raisi, M; Pourkhabbaz, HR; Banaee, M; Pourkhabbaz, A and Javanmardi, S. | 2018 | Iran | Lab-Based Study | Yes | Yes |
| Effects of saline conditions and hydrologic permanence on snail assemblages in wetlands of Northeastern China. | Wu, H; Guan, Q; Ma, H; Xue, Z; Yang, M and Batzer, DP | 2019 | China | Small-Scale Survey | No | No |
| Effects of Selection and Drift on G Matrix Evolution in a Heterogeneous Environment: A Multivariate Q(st)-F-st Test With the Freshwater Snail Galba truncatula | Chapuis, E; Martin, G and Goudet, J | 2008 | Switzerland | Lab-Based Study | No | No |
| Endemic occurrence of Fasciola hepatica in an alpine ecosystem, Pyrenees, Northeastern Spain | Roldan, C; Begovoeva, M; Lopez-Olvera, JR; Velarde, R; Cabezon, O; Min, ARM; Pizzato, F; Pasquetti, M; Aguilar, XF; Mentaberre, G; Serrano, E; Ribas, MP; Espunyes, J; Castillo-Contreras, R; Estruch, J and Rossi, L | 2021 | Spain | Small-Scale Survey | No | No |
| Environmental and biotic factors affecting freshwater snail intermediate hosts in the Ethiopian Rift Valley region | Olkeba, BK; Boets, P; Mereta, ST; Yeshigeta, M; Akessa, GM; Ambelu, A and Goethals, PL | 2020 | Ethiopia | Large-Scale Survey | No | No |
| Epidemiological studies on Fasciola hepatica in Gafsa oases (South West of Tunisia) | Hammami, H; Hamed, N and Ayadi, A | 2007 | Tunisia | Small-Scale Survey | Yes | Yes |
| Equines as reservoirs of human fascioliasis: transmission capacity, epidemiology and pathogenicity in Fasciola hepatica-infected mules | y Sierra, RM; Neira, G; Bargues, MD; Cuervo, PF; Artigas, P; Logarzo, L; Cortiñas, G; Ibaceta, DE; Garrido, AL; Bisutti, E and Mas-Coma, S | 2020 | Argentina | Field and Lab Study | Yes | No |
| Evolutionary implications of a high selfing rate in the freshwater snail Lymnaea truncatula. | Trouve, S; Degen, L; Renaud, F and Goudet J | 2003 | Switzerland | Lab-Based Study | No | No |
| Experimental colonization of new habitats by Galba truncatula O.F. Muller (Gastropoda : Lymnaeidae) in central France and their susceptibility to experimental infection with the trematode Fasciola hepatica L. | Vareille-Morel, C; Rondelaud, D and Dreyfuss, G | 2002 | France | Field Experiment | Yes | Yes |
| Fasciola hepatica and lymnaeld snails occurring at very high altitude in South America | Mas-Coma, S; Funatsu, IR and Bargues, MD | 2001 | Bolivia, Spain, Morocco, Portugal, Switzerland and France | Lab-Based Study | No | No |
| Fasciola hepatica and Paramphistomum daubneyi:changes in prevalences of natural infections in cattle and in Lymnaea truncatula from central France over the past 12 years | Mage, C; Bourgne, H; Toullieu, JM; Rondealud, D and Dreyfuss, G | 2002 | France | Large-Scale Survey | No | No |
| Fasciola hepatica infections in cattle and the freshwater snail Galba truncatula from Dakhla Oasis, Egypt | Arafa, WM; Hassan, AI; Snousi, SAM; El-Dakhly, KM; Holman, PJ; Craig, TM and Aboelhadid, SM | 2018 | Egypt | Small-Scale Survey | Yes | No |
| Fasciola hepatica infections in livestock flock, guanacos and coypus in two wildlife reserves in Argentina | Issia, L; Pietrokovsky, S; Sousa-Figueiredo, J; Stothard, JR and Wisnivesky-Colli, C | 2009 | Argentina | Small-Scale Survey | Yes | No |
| Fasciola species and their vertebrate and snail intermediate hosts in East and Southern Africa: a review | Malatji, MP; Pfukenyi, DM and Mukaratirwa, S | 2020 | Ethiopia, Tanzania, Uganda, Lesotho and South Africa | Literature Review | No | No |
| Fascioliasis in Llama, Lama glama, in Andean Endemic Areas: Experimental Transmission Capacity by the High Altitude Snail Vector Galba truncatula and Epidemiological Analysis of Its Reservoir Role | Mas-Coma, S; Cafrune, MM; Funatsu, IR, Mangold, AJ; Angles, R; Buchon, P; Fantozzi, MC; Artigas, P; Valero, MA and Bargues, MD | 2021 | Bolivia | Field and Lab Study | No | No |
| Fauna of Mollusks of the Lower Reaches of the Danube River within the Territory of Ukraine | Kornyushin, AV and Liashenko, AV | 2004 | Ukraine | Large-Scale Survey | No | No |
| Field and experimental evidence of preferential selfing in the freshwater mollusc Lymnaea truncatula (Gastropoda, Pulmonata) | Meunier, C; Hurtrez-Boussès, S; Jabbour-Zahab, R; Durand, P; Rondelaud, D and Renaud, F | 2004 | France | Lab-Based Study | Yes | Yes |
| Fine-scale mapping of vector habitats using very high resolution satellite imagery: a liver fluke case-study | De Roeck, E; Van Coillie, F; De Wulf, R; Soenen, K; Charlier, J; Vercruysse, J; Hantson, W; Ducheyne, E and Hendrickx, G | 2014 | Belgium | Small-Scale Survey | No | No |
| First investigations on the populations or Galba truncatula (Mollusca Gastropoda : Lyrunacidae), the snail host of Fasciola hepatica, in the irrigated district of Doukkala (northwestern Morocco). | Belfaiza, M; Moncef, M and Rondelaud, D | 2005 | Morocco | Large-Scale Survey | Yes | Yes |
| First report of Fasciola larva infection in Galba truncatula (Müller, 1774)(Gastropoda, Lymnaeidae) occurring in the natural environment in Hokkaido, Japan. | Ohari, Y; Hayashi, K; Mohanta, UK; Kuwahara, Y and Itagaki, T. | 2017 | Japan | Small-Scale Survey | Yes | No |
| First studies on the habitats of Galba truncatula (Mollusca Gastropoda : Lymnaeidae), the snail host of Fasciola hepatica, and the dynamics of snail populations in Northeastern Algeria | Mekroud, A; Benakha, A; Benlatreche, C; Rondelaud, D and Dreyfuss, G | 2002 | Algeria | Small-Scale Survey | Yes | Yes |
| Freshwater gastropods of the western part of the Kola Peninsula and northern Karelia (northern Europe) | Nekhaev, IO | 2021 | Russia | Large-Scale Survey | Yes | Yes |
| Freshwater mollusc biodiversity and conservation in two stressed Mediterranean basins | Perez-Quintero, JC | 2011 | Spain | Large-Scale Survey | Yes | No |
| Freshwater molluscs in mountain lakes of the Eastern Alps (Austria): relationship between environmental variables and lake colonization | Sturm, R. | 2007 | Austria | Small-Scale Survey | Yes | Yes |
| Freshwater molluscs of Kyrgyzstan with description of one new genus and species (Mollusca: Gastropoda) | Gloer, P; Boeters, HD and Pesic, V. | 2014 | Kyrgyzstan | Small-Scale Survey | Yes | Yes |
| Freshwater Snail Communities and Lake Classification. An Example from the Aland Islands, Southwestern Finland | Carlsson, R | 2001 | Finland | Small-Scale Survey | Yes | Yes |
| Freshwater snails (Mollusca: Gastropoda) of Bulgaria: an updated annotated checklist. | Georgiev, D and Hubenov, Z | 2013 | Bulgaria | Literature Review | No | No |
| Freshwater snails as the intermediate host of trematodes in Iran: a systematic review | Dodangeh, S; Daryani, A; Sharif, M; Gholami, S; Kialashaki, E; Moosazadeh, M and Sarvi, S | 2019 | Iran | Literature Review | No | No |
| Further Observations on the Life-history of Limnaea truncatula | Walton, CL; Jones, WN | 1926 | United Kingdom | Small-Scale Survey | No | No |
| Galba truncatula (Gastropoda, Lymnaeidae): effects of daily waterlevel variations on the ecology and ethology of populations living upstream from a dam | Hourdin, P; Vignoles, P; Dreyfuss, G and Rondelaud, D | 2006 | France | Small-Scale Survey | Yes | Yes |
| Galba truncatula (Gastropoda, Lymnaeidae): First findings on populations showing a single annual generation in lowland zones of central France | Rondelaud, D; Hourdin, P; Vignoles, P and Dreyfuss, G | 2009 | France | Small-Scale Survey | Yes | Yes |
| Galba truncatula (Mollusca Gastropoda, Lymnaeidae), the snail host of Fasciola hepatica: Its ability to withstand a periodic drying of its habitat in an irrigated area tinder a semiarid climate | Belfaiza, M; Moncef, M; Vignoles, P and Rondelaud, D | 2009 | Morocco | Field and Lab Study | Yes | Yes |
| Galba truncatula (Muller, 1774). A factor for spreading of fasciolosis. | Radev, V; Hrusanov D; Georgieva, K; and Bankov, I | 2008 | Bulgaria | Small-Scale Survey | No | No |
| Galba truncatula (OF Muller, 1774) (Gastropoda, Lymnaeidae): the colonization of new stations on acid soil by low numbers of snails | Dreyfuss, G; Vignoles, P and Rondelaud, D | 2021 | France | Field Experiment | Yes | Yes |
| Galba truncatula and Fasciola hepatica: Genetic costructures and interactions with intermediate host dispersal | Correa, AC; De Meeûs, T; Dreyfuss, G; Rondelaud, D. and Hurtrez-Boussès, S | 2017 | France | Field and Lab Study | Yes | No |
| Galba truncatula and Omphiscola glabra (Gastropoda, Lymnaeidae): present decline in populations living on sedimentary soils in central France | Dreyfuss, G; Vignoles, P and Rondelaud, D | 2017 | France | Large-Scale Survey | Yes | Yes |
| Gastropod Species Distribution and its Relation with some Physico-chemical Parameters of the Malatya's Streams (East Anatolia, Turkey) | Sahin, SK | 2012 | Turkey | Small-Scale Survey | Yes | Yes |
| Genetic uniformity, geographical spread and anthropogenic habitat modifications of lymnaeid vectors found in a One Health initiative in the highest human fascioliasis hyperendemic of the Bolivian Altiplano | Bargues, MD; Artigas, P; Angles, R; Osca, D; Duran, P; Buchon, P; Gonzales-Pomar, RK; Pinto-Mendieta, J and Mas-Coma, S | 2020 | Bolivia | Field and Lab Study | Yes | No |
| Growth rate of the intermediate snail host Galba truncatula influences redial development of the trematode Fascioloides magna | Rondelaud, D; Novobilsky, A; Hoglund, J; Kasny, M; Pankrac, J; Vignoles, P and Dreyfuss, G | 2014 | France | Lab-Based Study | Yes | Yes |
| Growth, Laying, and Survival Rates of the Galba truncatula Snails Infected with Fasciola hepatica | Aimeur, F; Mekroud, M; Titi, A; Ouchene, N and Mekroud, A | 2022 | Algeria | Field and Lab Study | No | No |
| Habitat suitability modelling for predicting potential habitats of freshwater snail intermediate hosts in Omo-Gibe river basin, Southwest Ethiopia | Yigezu, G; Mandefro, B; Mengesha, Y., Yewhalaw, D; Beyene, A; Ahmednur, M; Abdie, Y; Kloos, H and Mereta, ST | 2018 | Ethiopia | Large-Scale Survey | Yes | Yes |
| Habitats of Limnaea truncatula in England and Wales during Dry Seasons | Peters, BG | 1938 | United Kingdom | Small-Scale Survey | Yes | Yes |
| Habitats of Lymnaea-Truncatula intermediate host of Fasciola hepatica | Moens, R | 1981 | Belgium | Large-Scale Survey | Yes | No |
| High quantitative and no molecular differentiation of a freshwater snail (Galba truncatula) between temporary and permanent water habitats | Chapuis, E; Trouve, S; Facon, B; Degen, L and Goudet, J | 2007 | Switzerland | Lab-Based Study | Yes | Yes |
| Highland populations of Lymnaea truncatula infected with Fasciola hepatica survive longer under experimental conditions than lowland ones | Vignoles, P., Favennec, L., Dreyfuss, G. and Rondelaud, D. | 2002 | France and Peru | Lab-Based Study | No | No |
| Hydrobiological particularities of Maglavit Lake (Romania)–the place and role of Gastropod populations | Olivia, C | 2015 | Romania | Small-Scale Survey | Yes | Yes |
| Hydrographic network structure and population genetic differentiation in a vector of fasciolosis, Galba truncatula | Hurtrez-Boussès, S; Hurtrez, JE;Turpin, H; Durand, C; Durand, P; De Meeüs, T; Meunier, C; and Renaud, F | 2010 | France | Lab-Based Study | Yes | Yes |
| Identification of factors associated with Fasciola hepatica infection risk areas on pastures via an environmental DNA survey of Galba truncatula distribution using droplet digital and quantitative real-time PCR assays | Jones, RA; Davis, CN; Nalepa‐Grajcar, J; Woodruff, H; Williams, HW; Brophy, PM; and Jones, E | 2024 | United Kingdom | Small-Scale Survey | Yes | No |
| Identification of viable fluke metacercarial challenge to livestock | Cuthill, G | 2020 | United Kingdom | Small-Scale Survey | Yes | Yes |
| Influence of aestivation on the survival of Galba truncatula (Mollusca : Gasteropoda) populations according to altitude | Goumghar, MD; Rondelaud, D; Dreyfuss, G and Benlemlih, M | 2001 | France | Field and Lab Study | Yes | Yes |
| Interaction between the intermediate host of Fascioliasis in Tunisia, Galba truncatula and a possible competitor, Melanoides tuberculata (Muller, 1774): a field study | Ghouaidia, N. and Hammami, H. | 2014 | Tunisia | Small-Scale Survey | Yes | Yes |
| Lack of molluscan host diversity and the transmission of an emerging parasitic disease in Bolivia | Meunier, C; Tirard, C; Hurtrez‐Bousses, S; Durand, P; Bargues, MD; Mas‐Coma, S; Pointier, JP; Jourdane, J. and Renaud, F. | 2001 | Bolivia, France, Morocco, Spain and Portugual | Field and Lab Study | No | No |
| Larval trematode infections in Galba truncatula (Gastropoda, Lymnaeidae) from the Brenne Regional Natural Park, central France | Rondelaud, D; Vignoles, P and Dreyfuss, G | 2016 | France | Large-Scale Survey | Yes | Yes |
| Les réseaux de drainage superficiel et leur colonisation par Lymnaea truncatula Müller. A propos de quatre années d'observations en Haute-Vienne, France. In Annales de Recherches Vétérinaires | Rondelaud, D | 1983 | France | Field Experiment | Yes | No |
| Life history and population fluctuations of Lymnaea truncatula (Mull), the snail vector of fascioliasis. | Heppleston, P.B | 1972 | United Kingdom | Small-Scale Survey | Yes | Yes |
| Life history traits variation in heterogeneous environment: The case of a freshwater snail resistance to pond drying | Chapuis, E and Ferdy, JB | 2011 | Switzerland | Lab-Based Study | No | No |
| Liver Rot of Sheep, and Bionomics of Limnaea truncatula in the Aberystwyth Area | Walton, CL | 1918 | United Kingdom | Large-Scale Survey | Yes | No |
| Local adaptation of the trematode Fasciola hepatica to the snail Galba truncatula | Dreyfuss, G; Vignoles, P and Rondelaud, D | 2012 | France | Field and Lab Study | Yes | Yes |
| Longitudinal study on the temporal and micro-spatial distribution of Galba truncatula in four farms in Belgium as a base for small-scale risk mapping of Fasciola hepatica | Charlier, J; Soenen, K; De Roeck, E; Hantson, W; Ducheyne, E; Van Coillie, F; De Wulf, R; Hendrickx, G and Vercruysse, J | 2014 | Belgium | Small-Scale Survey | Yes | Yes |
| Lymnaea schirazensis, an Overlooked Snail Distorting Fascioliasis Data: Genotype, Phenotype, Ecology, Worldwide Spread, Susceptibility, Applicability | Bargues, MD; Artigas, P; Khoubbane, M; Flores, R Gloer, P; Rojas-Garcia, R; Ashrafi, K; Falkner, G and Mas-Coma, S | 2011 | Not Stated | Lab-Based Study | No | No |
| Lymnaea truncatula MULLER, 1774 (Pulmonata: Lymnaeidae) infected with Fasciola hepática (Linnaeus, 1758)(Trematoda: Digenea), in Moscow districts, Russian Federation. | Villavicencio Á; Gorochov, V and Vasconcellos, MCD | 2006 | Russia | Small-Scale Survey | Yes | No |
| Lymnaea truncatula, intermediate host of some Plagiorchiidae and Notocotylidae species in Leon, NW Spain | Manga-Gonzalez, Y; González-Lanza, C and Kanev, I | 1994 | Spain | Small-Scale Survey | No | Yes |
| Lymnaeid habitats in swampy meadows on acid soil: Effects of agricultural impacts. | Vareille, L; Vareille-Morel, C; Dreyfuss, G and Rondelaud, D. | 1996 | France | Small-Scale Survey | Yes | Yes |
| Lymnaeidae from Santander and bordering departments of Colombia: Morphological characterization, molecular identification and natural infection with Fasciola hepatica | Pereira, AE; Uribe, N and Pointier, JP | 2020 | Colombia | Large-Scale Survey | No | No |
| Lymnea truncatula (Mollusca Lymnaeidae) in the rice fields of eastern Spain. | Valero, MA; Marti, R; Marcos, MD; Robles, F and Mas-Coma, S | 1998 | Spain | Small-Scale Survey | Yes | No |
| Malacofauna of Svityaz and Pisochne Lakes in Shatsk National Nature Park and the role of molluscs in formation of cercarial dermatitis nidi | Koltun, I.O; Liesnik, VV and Khamar, IS | 2020 | Ukraine | Small-Scale Survey | Yes | Yes |
| Malacological survey in a bottle of water: A comparative study between manual sampling and environmental DNA metabarcoding approaches. | Mulero, S; Toulza, E; Loisier, A; Zimmerman, M; Allienne, JF; Foata, J; Quilichini, Y; Pointier, JP Rey, O and Boissier, J | 2021 | France | Small-Scale Survey | Yes | Yes |
| Metal bioaccumulation, oxidative stress, and biochemical alterations in the freshwater snail (Galba truncatula) exposed to municipal sewage | Banaee, M & Taheri, S | 2019 | Iran | Lab-Based Study | Yes | Yes |
| Microsatellites in the hermaphroditic snail, Lymnaea truncatula, intermediate host of the liver fluke, Fasciola hepatica | Trouvé, S; Degen, L; Meunier, C; Tirard, C; Hurtrez-Bousses, S; Durand, P; Guégan, JF; Goudet J and Renaud, F | 2000 | Switzerland | Lab-Based Study | No | No |
| Molecular characterisation of Galba truncatula, Lymnaea neotropica and L. schirazensis from Cajamarca, Peru and their potential role in transmission of human and animal fascioliasis | Bargues, MD; Artigas, P; Khoubbane, M; Ortiz, P; Naquira, C and Mas-Coma, S | 2012 | Peru | Field and Lab Study | No | No |
| Molecular characterization of cryptic and sympatric lymnaeid species from the Galba/Fossaria group in Mendoza Province, Northern Patagonia, Argentina | Standley, CJ; Prepelitchi, L; Pietrokovsky, SM; Issia, L; Stothard, JR and Wisnivesky-Colli, C | 2013 | Argentina | Field and Lab Study | No | No |
| Molecular detection of the infection with Fasciola hepatica in field-collected snails of Galba truncatula and Lymnaea stagnalis from West Azarbaijan, Iran. | Malekzadeh-Viayeh, R; Imani Baran, A and Yakhchali, M | 2015 | Iran | Field and Lab Study | Yes | No |
| Molecular Evolution of Freshwater Snails with Contrasting Mating Systems | Burgarella, C; Gayral, P., Ballenghien, M; Bernard, A; David, P; Jarne, P; Correa, A; Hurtrez-Boussès, S; Escobar, J; Galtier, N and Glémin, S | 2015 | Not Stated | Lab-Based Study | No | No |
| Mollusc communities along upstream–downstream gradients in small coastal basins of the south-western Iberian Peninsula | Pérez-Quintero, JC | 2013 | Portugal and Spain | Small-Scale Survey | Yes | Yes |
| Molluscs of selected watercourses and reservoirs in Vilnius | Wlosik-Bienczak, E | 2005 | Lithuania | Small-Scale Survey | Yes | No |
| Mollusks (Gastropoda) as intermediate hosts of cattles’ trematodes (Trematoda) in conditions of Dnipro basin’s small ponds (Northern Ukraine) | Feshchenko, DV; Bakhur, TI; Selcuk, BH; Antipov, AA; Zghozinska, OA; Dubova, OA; Yevstafyeva, VO; Goncharenko, VP; Shahanenko, RV; Shahanenko, VS and Melnychuk, VV | 2019 | Ukraine | Small-Scale Survey | Yes | No |
| Mollusks biodiversity of Lake Sevan, Armenia | Mashkova, IV; Krupnova, TG; Kostryukova, AM; Harutyunova, LJ; Varuzhan, HS and Vlasov, NE | 2018 | Armenia | Small-Scale Survey | Yes | Yes |
| Natural infection of Lymnaea truncatula by the liver fluke Fasciola hepatica in the Porma Basin, León, NW Spain | Manga-González, Y; Gonzalez-Lanza, C and Otero-Merino, CB | 1991 | Spain | Small-Scale Survey | Yes | No |
| New land snails from Montenegro and Albania | Subai, P | 2009 | Montenegro | Small-Scale Survey | Yes | Yes |
| Non-marine aquatic molluscs (Gastropoda, Bivalvia) of Rab Island (Croatia) | Beran, L | 2015 | Croatia | Small-Scale Survey | Yes | Yes |
| Notes on the continental malacofauna of Rhodes, with two new species for the fauna of the island. | Páll-Gergely, B and Csabai, Z | 2008 | Greece | Small-Scale Survey | Yes | No |
| Notes on the ecology and species diversity of the inland molluscs of Samothraki Island (North-Eastern Greece) | Georgiev, D and Stoycheva, S | 2010 | Greece | Small-Scale Survey | No | No |
| Occurrence of Fasciola (Digenea: Fasciolidae) Species in Livestock, Wildlife and Humans, and the Geographical Distribution of Their Intermediate Hosts in South Africa-A Scoping Review | Nyagura, I Malatji, MP and Mukaratirwa, S | 2022 | South Africa | Literature Review | Yes | No |
| One Health initiative in the Bolivian Altiplano human fascioliasis hyperendemic area: Lymnaeid biology, population dynamics, microecology and climatic factor influences | Bargues, MD; Angles, R; Coello, J; Artigas, P; Funatsu, IR; Cuervo, PF; Buchon, P and Mas-Coma, S | 2021 | Bolivia | Field and Lab Study | Yes | Yes |
| Patterns of Fasciola hepatica infection in Danish dairy cattle: implications for on-farm control of the parasite based on different diagnostic methods | Takeuchi-Storm, N; Denwood, M; Petersen, HH; Enemark, HL; Stensgaard, AS; Sengupta, ME; Beesley, NJ; Hodgkinson, J; Williams, D and Thamsborg, SM | 2018 | Denmark | Small-Scale Survey | Yes | Yes |
| Phylogeography and genetic divergence of some lymnaeid snails, intermediate hosts of human and animal fascioliasis with special reference to lymnaeids from the Bolivian Altiplano | Jabbour-Zahab, R; Pointier, JP; Jourdane, J; Jarne, P; Oviedo, JA; Bargues, MD; Mas-Coma, S; Anglés, R; Perera, G; Balzan, C. and Khallayoune, K | 1997 | Bolivia, France, Portugal and Morocco | Lab-Based Study | No | No |
| Physico-chemical parameters and benthic macroinvertebrates of Ogunpa river at Bodija, Ibadan, Oyo State. | Efe, O; Kokoette, EI and Alex, UAA | 2012 | Nigeria | Small-Scale Survey | Yes | Yes |
| Potential Hybridization of Fasciola hepatica and F. gigantica in Africa-A Scoping Review | Nukeri, S; Malatji, MP; Sengupta, ME; Vennervald, BJ; Stensgaard, AS; Chaisi, M and Mukaratirwa, S | 2022 | Algeria, Egypt, Tunisia, Morocco, Lesotho, South Africa, Ethopia, Tanzania and Uganda | Literature Review | No | No |
| Prevalence of Fasciola hepatica in Galba truncatula detected by Multiplex PCR in the province of El Tarf (Algeria) | Righi, S; Benakhla, A; Mekroud, A; Ouchene, N and Sedraoui, S | 2016 | Algeria | Lab-Based Study | Yes | No |
| Prevalence of Fasciola hepatica in the intermediate host Lymnaea truncatula detected by real time TaqMan PCR in populations from 70 Swiss farms with cattle husbandry | Schweizer, G; Meli, ML; Torgerson, PR; Lutz, H; Deplazes, P and Braun, U. | 2007 | Switzerland | Large-Scale Survey | Yes | Yes |
| Prevalence of Fascioloides magna in Galba truncatula in the Danube backwater area east of Vienna, Austria. | Hörweg, C; Prosl, H; Wille-Piazzai, W; Joachim, A and Sattmann, H. | 2011 | Austria | Large-Scale Survey | Yes | Yes |
| Radular variations in freshwater snails of the family Lymnaeidae (Mollusca: Gastropoda: Basommatophora) from northwestern Iran | Yakhchali, M and Deilamy, LJ | 2012 | Iran | Lab-Based Study | No | No |
| Rapid detection of Galba truncatula in water sources on pasture-land using loop-mediated isothermal amplification for control of trematode infections | Davis, CN; Tyson, F; Cutress, D; Davies, E; Jones, DL; Brophy, PM; Prescott, A; Rose, MT; Williams, M; Williams, HW and Jones, RA | 2020 | United Kingdom | Field Experiment | Yes | Yes |
| Real-time PCR strategy for rapid discrimination among main lymnaeid species from Argentina | Duffy, T; Kleiman, F; Pietrokovsky, S; Issia, L; Schijman, AG and Wisnivesky-Colli, C | 2009 | Argentina | Lab-Based Study | No | No |
| Recovery of Fascioloides magna (Digenea) population in spite of treatment programme? Screening of Galba truncatula (Gastropoda, Lymnaeidae) from Lower Austria | Haider, M; Hörweg, C; Liesinger, K; Sattmann, H and Walochnik, J | 2012 | Austria | Small-Scale Survey | Yes | No |
| Relationship between individual metabolic rate and patch departure behaviour: evidence from aquatic gastropods | Cozzoli, F; Shokri, M; Ligetta, G; Ciotti, M; Gjoni, V; Marrocco, V; Vignes, F and Basset, A | 2020 | Italy | Lab-Based Study | No | No |
| Relationships between the amphibious behaviour of the snail Galba truncatula (Mollusca Gastropoda : Lymnaeidae), altitude, and the type of its habitats in central Morocco | Goumghar, MD; Vignoles, P; Rondelaud, D; Dreyfuss, G and Benlemlih, M | 2004 | Morocco | Small-Scale Survey | Yes | Yes |
| Relationships between the distribution of Galba truncatula (Gastropoda: Lymnaeidae) climatic conditions and the altitude of municipalities in Haute Vienne (France) | Dreyfuss, G; Vignoles, P and Rondelaud, D | 2018 | France | Large-Scale Survey | Yes | Yes |
| Ribosomal DNA ITS-1 sequencing of Galba truncatula (Gastropoda, Lymnaeidae) and its potential impact on fascioliasis transmission in Mendoza, Argentina | Bargues, MD; y Sierra, RM; Gómez, HG; Artigas, P. and Mas-Coma, S. | 2006 | Argentina, Morocco, Bolivia, Spain, Potugal, Switzerland and France | Lab-Based Study | No | No |
| Schistosomatidae from the trematode fauna of aquatic and semi-aquatic birds in Uzbekistan | Akramova, F; Shakarbaev, U; Arepbaev, I; Yorkulov, Z; Ravshanova, A; Saidova, S and Azimov, D | 2023 | Uzbekistan | Small-Scale Survey | No | No |
| Scientific Results of the Yale North India Expedition. Biological Report No. 21 Aquatic and Amphibious Molluscs | Prashad, B | 1937 | India | Small-Scale Survey | No | No |
| Seasonal variations in the microclimate of Lymnaea truncatula habitats | Smith, G and Wilson, RA | 1980 | United Kingdom | Small-Scale Survey | No | No |
| Seasonal variations of Fasciola hepatica infection in goats in the area of Haouz (Morocco). | Khallaayoune, K and El Hari, M | 1991 | Morocco | Small-Scale Survey | No | No |
| Semi-naturally managed forests support diverse land snail assemblages in Estonia | Remm, L and Lõhmus, A | 2016 | Estonia | Large-Scale Survey | No | No |
| Small effective population sizes in a widespread selfing species, Lymnaea truncatula (Gastropoda: Pulmonata) | Meunier, C; Hurtrez‐Bousses, S; Durand, P; Rondelaud, D and Renaud, F | 2004 | France | Lab-Based Study | Yes | Yes |
| Soil type and the distribution of Lymnaea truncatula. | Wright, PS, and Swire, PW | 1984 | United Kingdom | Small-Scale Survey | Yes | No |
| Spatio-temporal distribution of freshwater snail species in relation to migration and environmental factors in an irrigated area from Morocco | Chlyeh, G; Dodet, M; Delay, B; Khallaayoune, K and Jarne, P | 2005 | Morocco | Small-Scale Survey | Yes | Yes |
| Species composition of freshwater snail communities in lakes of southern and western Finland | Aho, J; Ranta, E; and Vuorinen, J | 1981 | Finland | Small-Scale Survey | Yes | Yes |
| Species-area relationships, water chemistry and species turnover of freshwater snails on the Åland Islands, southwestern Finland. | Carlsson, R | 2002 | Finland | Small-Scale Survey | No | No |
| Sporadic notes on the land snail (Gastropoda) fauna of the North Bihor Mountains, Romania | Domokos, T and Páll-Gergely, B | 2021 | Romania | Small-Scale Survey | Yes | Yes |
| Spread of the fascioliasis endemic area assessed by seasonal follow-up of rDNA ITS-2 sequenced lymnaeid populations in Cajamarca, Peru | Bardales-Valdivia, JN; Bargues, MD; Hoban-Vergara, C; Bardales-Bardales, C; Goicochea-Portal, C; Bazan-Zurita, H; Del Valle-Mendoza, J; Ortiz, P and Mas-Coma, S. | 2021 | Peru | Small-Scale Survey | Yes | No |
| Standard faunistical work on the molluscs of Codru-Moma Mountains (Romania). | Domokos, T and Lennert, J | 2007 | Romania | Small-Scale Survey | Yes | Yes |
| Studies on the Life-Cycle of Fasciola Hepatica (Linnaeus) and of Its Snail Host, Limnaea (Galba) Truncatula (Müller), in the Field and Under Controlled Conditions in the Laboratory | Roberts, EW | 1950 | United Kingdom | Field and Lab Study | Yes | Yes |
| Study on the Vector Role for Calicophoron daubneyi of Some Aquatic Snails form Western Romania | Sîrbu, CB; Pet, I; Morariu, F; Goina CA; Morariu, MM; Sîrbu; BAM and Morariu, S | 2005 | Romania | Small-Scale Survey | No | No |
| Sub-lethal effects of dimethoate alone and in combination with cadmium on biochemical parameters in freshwater snail, Galba truncatula | Banaee, M; Sureda, A; Taheri, S; and Hedayatzadeh, F | 2019 | Iran | Lab-Based Study | Yes | No |
| Survey and population dynamics of freshwater snails in newly settled areas of the Sinai Peninsula. | El-Kady, GA; Shoukry, A; Reda LA and El-Badri YS | 2000 | Egypt | Small-Scale Survey | No | No |
| Synopsis of the Egyptian freshwater snail fauna | Lotfy, WM and Lotfy, LM | 2015 | Egypt | Literature Review | No | No |
| Systematic descriptions and seasonal variations of mollusc in Chandigarh (U.T., India) and its surrounding freshwater bodies | Maansi, RJ and Wats, M | 2021 | India | Small-Scale Survey | Yes | Yes |
| Temporal dynamics of trematode intermediate snail host environmental DNA in small water body habitats | Jones, RA; Davis, CN; Jones, DL; Tyson, F; Davies, E; Cutress, D; Brophy, PM; Rose, MT; Williams, M; and Williams, HW | 2021 | United Kingdom | Small-Scale Survey | Yes | Yes |
| Temporal studies on Fasciola hepatica in Galba truncatula in the west of Ireland | Relf, V; Good, B; Hanrahan, JP; McCarthy, E; Forbes, AB and Dewaal, T | 2011 | Republic of Ireland | Small-Scale Survey | Yes | Yes |
| The characteristics of habitats colonized by three species of Lymnaea (Mollusca) in swampy meadows on acid soil: their interest for control of fasciolosis | Vareille-Morel, C., Dreyfuss, G. and Rondelaud, D | 1999 | France | Large-Scale Survey | Yes | Yes |
| The contamination of wild watercress with Fasciola hepatica in central France depends on the ability of several lymnaeid snails to migrate upstream towards the beds | Rondelaud, D; Hourdin, P; Vignoles, P and Dreyfuss, G | 2005 | France | Field Experiment | Yes | Yes |
| The control of Galba truncatula (Gastropoda: Lymnaeidae) by the terrestrial snail Zonitoides nitidus on acid soils | Rondelaud, D; Vignoles, P; Dreyfuss, G and Mage, C | 2006 | France | Field Experiment | Yes | Yes |
| The detection of snail host habitats in liver fluke infected farms by use of plant indicators | Rondelaud, D; Hourdin, P; Vignoles, P; Dreyfuss, G and Cabaret, J | 2011 | France | Field Experiment | Yes | Yes |
| The distribution of Fasciola hepatica and Fasciola gigantica within southern Tanzania - constraints associated with the intermediate host | Walker, SM; Makundi, AE; Namuba, FV; Kassuku, AA; Keyyu, J; Hoey, EM; Prödohl, P; Stothard, JR and Trudgett, A. | 2008 | Tanzania | Field and Lab Study | Yes | Yes |
| The ecological complex role of the macroinvertebrate fauna from the River Ciric (Iași, România). | Nicoară, M; Erhan, M; Plăvan, G; Cojocaru, I; Davideanu, A and Nicoară, A | 2009 | Romania | Small-Scale Survey | No | No |
| The effects of low temperature on Lymnaea truncatula. | Hodasi, JKM | 1976 | United Kingdom | Lab-Based Study | No | No |
| The finding of North American freshwater gastropods of the genus Planorbella Haldeman, 1842 (Pulmonata: Planorbidae) in East Siberia | Sitnikova, T; Soldatenko, E; Kamaltynov, R and Riedel, F | 2010 | Russia | Small-Scale Survey | Yes | Yes |
| The freshwater molluscs from the Târnava rivers basin (Transylvania, Romania). | Sîrbu, I | 2005 | Romania | Small-Scale Survey | Yes | Yes |
| The habitats of Lymnaea truncatula Muller (Mollusca) along two river banks | Dreyfuss, G; Vareille-Morel, V and Rondelaud, D | 1997 | France | Small-Scale Survey | Yes | Yes |
| The Life-History of Limnaea truncatula under Laboratory Conditions. | Kendall, SB | 1953 | United Kingdom | Lab-Based Study | No | No |
| The Mollusk Fauna of Istranca Stream (Terkos-Istanbul) And Some Physico-Chemical Parameters of Their Abundance | Şahin, SK; Dokumcu, D and Ozulug, O | 2017 | Turkey | Small-Scale Survey | Yes | Yes |
| The mollusk fauna of Lake Sapanca (Turkey: Marmara) and some physico-chemical parameters of their abundance. | Şahin, SK and Yildirim, MZ | 2007 | Turkey | Small-Scale Survey | Yes | Yes |
| The natural watercress beds in the region of Limousin (France). About of some observations on numerical variations of Lymnaeid snails. | Rondelaud, D and Mage, C | 1990 | France | Small-Scale Survey | Yes | Yes |
| The New Red List of the molluscs of Latvia | Rudzīte, M; Boikova, E; Dreijers, E; Jakubāne, I; Parele, E; Pilāte, D and Rudzītis, M | 2018 | Latvia | Literature Review | No | No |
| The Northern Bolivian Altiplano: a region highly endemic for human fascioliasis | Mas‐Coma, S; Angles, R; Esteban, JG; Bargues, MD; Buchon, P; Franken, M and Strauss, W | 1999 | Bolivia | Small-Scale Survey | Yes | No |
| The performance of a PCR assay for field studies on the prevalence of Fasciola hepatica infection in Galba truncatula intermediate host snails | Kozak, M and Wedrychowicz, H | 2010 | Poland | Lab-Based Study | No | No |
| The prevalence and development of digenean parasites within their intermediate snail host, Galba truncatula, in a geographic area where the presence of Calicophoron daubneyi has recently been confirmed | Jones, RA; Williams, HW; Dalesman, S; Ayodeji, S; Thomas, RK and Brophy, PM | 2017 | United Kingdom | Small-Scale Survey | Yes | No |
| The relationship between the size of Lymnaea truncatula naturally infected with Fasciola hepatica and the intensity and maturity of the redial infection. | Smith, G | 1984 | United Kingdom | Small-Scale Survey | No | No |
| The studies of molluscs in moricsala strict nature reserve and other sites in western Latvia | Pilāte, D | 2013 | Latvia | Small-Scale Survey | No | No |
| The Worm Burden of Sheep on Improved and Unimproved Hill Pastures. | Rees, G | 1942 | United Kingdom | Small-Scale Survey | Yes | Yes |
| Towards assessing fine-scale indicators for the spatial transmission risk of Fasciola hepatica in cattle | Charlier, J; Bennema, SC; Caron, Y; Counotte, M; Ducheyne, E; Hendrickx, G and Vercruysse, J | 2011 | Belgium | Small-Scale Survey | Yes | No |
| Transmission of Calicophoron daubneyi and Fasciola hepatica in Galicia (Spain): Temporal follow-up in the intermediate and definitive hosts | Iglesias-Piñeiro, J; González-Warleta, M; Castro-Hermida, JA; Córdoba, M; González-Lanza, C; Manga-González, Y and Mezo, M | 2016 | Spain | Small-Scale Survey | Yes | Yes |
| Transmission patterns of Fasciola hepatica to ruminants in Sweden | Novobilsky, A; Engstrom, A; Sollenberg, S; Gustafsson, K; Morrison, DA and Hoglund, J | 2014 | Sweden | Small-Scale Survey | No | No |
| Validation of an interactive map assessing the potential spread of Galba truncatula as intermediate host of Fasciola hepaticain Switzerland | Baggenstos, R; Dahinden, T; Torgerson, PR; Bar, H; Rapsch, C and Knubben-Schweizer, G | 2016 | Switzerland | Small-Scale Survey | Yes | No |
| Variability of Fasciola infections in Lymnaea truncatula as a function of snail generation and snail activity. | Rondelaud, D and Dreyfuss, G | 1997 | France | Field and Lab Study | No | No |
| Vertical spatial behaviour patterns of Lymnaea truncatula in relation with origin of snails, infection with Fasciola hepatica, and experimental environment | Moukrim, A. and Rondelaud, D | 1992 | Morocco | Lab-Based Study | Yes | Yes |

**Supplementary Table S5:** Summary of information regarding pH of *Galba truncatula* habitats given by studies.

| **Study Title** | **Average pH** | **Minimum pH** | **Maximum pH** | **Trend** | **pH Source** |
| --- | --- | --- | --- | --- | --- |
| A Review of Species Diversity, Distribution and Ecology of Freshwater Gastropod Molluscs Inhabiting the Ukrainian Transcarpathian. | N/A | 6.8 | 8.5 | Not Stated or Calculated | Water |
| Aplexa hypnorum (Gastropoda: Physidae) exerts competition on two lymnaeid species in periodically dried ditches | N/A | 6.7 | 7.8 | Not Stated or Calculated | Water |
| Aquatic molluscs in high mountain lakes of the Eastern Alps (Austria): Species-environment relationships and specific colonization behaviour | N/A | 5.8 | 7.6 | No Preference or Neutral | Water |
| Bovine fasciolosis at increasing altitudes: Parasitological and malacological sampling on the slopes of Mount Elgon, Uganda | N/A | 5.6 | 9.4 | Not Stated or Calculated | Water |
| Changes in the Populations of Two Lymnaeidae and Their Infection by Fasciola hepatica and/or Calicophoron daubneyi over the Past 30 Years in Central France | N/A | 5.8 | 7 | Not Stated or Calculated | Water |
| Consequences of invasion by Pseudosuccinea columella on the dynamics of native lymnaeids living on the acid soils of central France | N/A | 5.6 | 7 | Not Stated or Calculated | Water |
| Current decline in the number and size of Galba truncatula and Omphiscola glabra populations, intermediate hosts of Fasciola hepatica, on the acidic soils of Central France | N/A | 5.6 | 7 | Not Stated or Calculated | Water |
| Decline in the number and size of populations of two Lymnaeidae living in central France over the last decade | N/A | 5.8 | 8.1 | Not Stated or Calculated | Water |
| Distribution of Mollusca fauna in the streams of Tunceli Province (East Anatolia, Turkey) and its relationship with some physicochemical parameters | 7.05 | 6.1 | 8.1 | Not Stated or Calculated | Water |
| Experimental colonization of new habitats by Galba truncatula O.F. Muller (Gastropoda : Lymnaeidae) in central France and their susceptibility to experimental infection with the trematode Fasciola hepatica L. | N/A | 5.8 | 7 | Not Stated or Calculated | Water |
| Freshwater mollusc biodiversity and conservation in two stressed Mediterranean basins | N/A | 7.4 | 8.5 | Possitive | Water |
| Freshwater molluscs in mountain lakes of the Eastern Alps (Austria): relationship between environmental variables and lake colonization | N/A | 5.8 | 7.6 | Not Stated or Calculated | Water |
| Galba truncatula (OF Muller, 1774) (Gastropoda, Lymnaeidae): the colonization of new stations on acid soil by low numbers of snails | 7.7 | 5.8 | 6.8 | Not Stated or Calculated | Water |
| Galba truncatula and Omphiscola glabra (Gastropoda, Lymnaeidae): present decline in populations living on sedimentary soils in central France | N/A | 6.8 | 8.1 | Not Stated or Calculated | Water |
| Gastropod Species Distribution and its Relation with some Physico-chemical Parameters of the Malatya's Streams (East Anatolia, Turkey) | N/A | 7.3 | 7.4 | Not Stated or Calculated | Water |
| Habitats of Limnaea truncatula in England and Wales during Dry Seasons | 7.5 | 6.9 | 8.2 | Not Stated or Calculated | Water |
| HABITATS OF LYMNAEA-TRUNCATULA, INTERMEDIATE HOST OF FASCIOLA-HEPATICA | N/A | 5.4 | 8.7 | No Preference or Neutral | Water |
| Hydrobiological particularities of Maglavit Lake (Romania)–the place and role of Gastropod populations | 7.2 | 6.5 | 8.5 | Not Stated or Calculated | Water |
| Identification of factors associated with Fasciola hepatica infection risk areas on pastures via an environmental DNA survey of Galba truncatula distribution using droplet digital and quantitative real-time PCR assays | N/A | N/A | N/A | No Preference or Neutral | Water |
| Influence of aestivation on the survival of Galba truncatula (Mollusca : Gasteropoda) populations according to altitude | N/A | 6.5 | 6.7 | Not Stated or Calculated | Water |
| Life history and population fluctuations of Lymnaea truncatula (Mull), the snail vector of fascioliasis. | N/A | 5.5 (Not Included) | 8.5 (Not Included) | Not Stated or Calculated | Not Stated |
| Local adaptation of the trematode Fasciola hepatica to the snail Galba truncatula | N/A | 6.9 | 7.6 | Not Stated or Calculated | Water |
| Longitudinal study on the temporal and micro-spatial distribution of Galba truncatula in four farms in Belgium as a base for small-scale risk mapping of Fasciola hepatica | N/A | N/A | N/A | Positive | Soil and Water |
| Molecular detection of the infection with Fasciola hepatica in field-collected snails of Galba truncatula and Lymnaea stagnalis from West Azarbaijan, Iran. | 6.5 | 5 | 9 | Not Stated or Calculated | Water |
| Mollusc communities along upstream–downstream gradients in small coastal basins of the south-western Iberian Peninsula | N/A | 7.5 | 8.7 | Not Stated or Calculated | Water |
| Natural infection of Lymnaea truncatula by the liver fluke Fasciola hepatica in the Porma Basin, León, NW Spain | N/A | 5.4 | 8.5 | Not Stated or Calculated | Water |
| One Health initiative in the Bolivian Altiplano human fascioliasis hyperendemic area: Lymnaeid biology, population dynamics, microecology and climatic factor influences | N/A | 5.5 | 8.5 | Negative | Water |
| Relationships between the distribution of Galba truncatula (Gastropoda: Lymnaeidae) climatic conditions and the altitude of municipalities in Haute Vienne (France) | N/A | 5.6 | 7.8 | Positive | Water |
| Temporal dynamics of trematode intermediate snail host environmental DNA in small water body habitats | 6.35 | N/A | N/A | Negative | Water |
| The characteristics of habitats colonized by three species of Lymnaea (Mollusca) in swampy meadows on acid soil: their interest for control of fasciolosis | N/A | 5.5 | 7.8 | Not Stated or Calculated | Water |
| The mollusk fauna of Lake Sapanca (Turkey: Marmara) and some physico-chemical parameters of their abundance. | 7.8 | 6.7 | 8.6 | Not Stated or Calculated | Water |
| The natural watercress beds in the region of Limousin (France). About of some observations on numerical variations of Lymnaeid snails. | N/A | 5.5 | 6.7 | Not Stated or Calculated | Water |
| The Northern Bolivian Altiplano: a region highly endemic for human fascioliasis | 7.7 | 6 | 9.4 | Not Stated or Calculated | Water |
